# Novel cVEMP procedure reveals sexual dimorphism in peak to trough latency

**DOI:** 10.3389/fnint.2025.1454924

**Published:** 2025-04-09

**Authors:** Max Gattie, Elena V. M. Lieven, Karolina Kluk

**Affiliations:** ^1^Department of Otolaryngology, Feinberg School of Medicine, Northwestern University, Chicago, IL, United States; ^2^Manchester Centre for Audiology and Deafness (ManCAD), The University of Manchester, Manchester, United Kingdom; ^3^The ESRC International Centre for Language and Communicative Development (LuCiD), The University of Manchester, Manchester, United Kingdom

**Keywords:** vestibular-evoked myogenic potential (VEMP), cVEMP, sex, dimorphism, vestibular

## Abstract

**Introduction:**

Sex difference in latency for cervical vestibular-evoked myogenic potential (VEMP) has been reported in Brown Norway rats. Human investigations of sex difference in VEMP latency have shown inconsistent results, although there are indicators of sexual dimorphism in vestibular function and a higher reporting rate for vestibular disorder in women than in men.

**Methods:**

Sex effects in human VEMP were re-evaluated here using a procedure adapting clinical protocols for higher sensitivity. VEMP was compared between 24 women and 24 men using a novel procedure that (1) controlled neck tension with biofeedback and a padded head bar; (2) used body-conducted stimuli to eliminate sound exposure concerns and collect appreciably more data than is feasible with air-conducted stimuli; which in turn (3) increased statistical power because there were sufficient data for a linear mixed effects regression modelling analysis.

**Results:**

Women had significantly shorter VEMP peak to trough latency than men. The sex difference of 2.4 ms (95% CI [−0.9, −3.9], p = 0.0020) was 21% of the mean 11.4 ms VEMP peak to trough latency measured across women and men. There was no significant sex difference in VEMP peak to trough amplitude. These findings are a reversal of several prior studies in humans, reviewed here with a simulation indicating the studies may have been underpowered.

**Discussion:**

Findings are consistent with those in Brown Norway Rats, for which a study design featuring a custom rodent holder to control neck tension, extension of test sequences in comparison to those typically used in VEMP protocols for humans, and insertion of electrodes subcutaneously will have increased sensitivity compared to that achievable with clinical VEMP protocols for humans. Findings are interpreted as sex hormones affecting myelination or synaptic response; sexual dimorphism in neck/head size may also have contributed. The vestibular periphery and brainstem are highly conserved across vertebrates with similar findings in rat and human supporting use of VEMP as a reliable, non-invasive indicator of vestibular function. VEMP measures in humans may require higher sensitivity than is achievable using current clinical protocols in order to produce consistent results.

## 1 Introduction

### 1.1 Background

**Figure 1:**
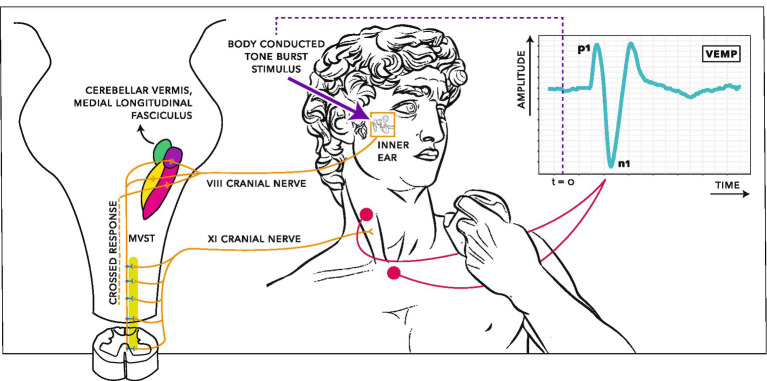
Electrophysiological recording arrangement for cervical vestibular-evoked myogenic potentials (VEMPs). The stimulus is a vibratory tone burst. Energy from the tone burst deflects hair cells in the vestibular system, creating an electrical impulse along the VIII cranial nerve. These vestibular afferents synapse in the vestibular nucleus then descend along the medial vestibulospinal tract (MVST). The MVST connects to the XI cranial nerve, which branches to synapse on motoneurons innervating the ipsilateral sternocleidomastoid. The brief activity from a tone burst results in a brief inhibition of tonic activity in the muscle fibres of the sternocleidomastoid. This inhibitory potential can be recorded between electrodes on the sternum and the belly of the sternocleidomastoid, creating a wave form with a characteristic peak (p1) and trough (n1) at approximately 13 ms and 23 ms after stimulus onset. The vestibulo-collic reflex also includes crossed and ascending components, not shown in detail. Based on Kim et al. (2010), Oh et al. (2016), and Colebatch et al. (2016). Creative Commons CC BY-NC-SA 4.0.

Although vertebrates can be found with a wide variety of adaptations across sensory organs specialised for hearing, such as the basillar papilla and cochlea, the consistency observed in their vestibular sensory input indicates a high degree of conservation in vestibular peripheral end organs, hindbrain vestibular nuclei and the associated projection patterns (Kopecky et al., 2011; Straka and Baker, 2013; Fritzsch et al., 2013; Fritzsch and Straka, 2014; Lipovsek and Wingate, 2018). Since its initial description in the 1990s (Colebatch and Halmagyi, 1992) the cervical vestibular-evoked myogenic potential (VEMP) has become accepted as a reliable test of vestibular function. It measures a short inhibition of tonic activity in the sternocleidomastoid muscle (SCM) (Corneil and Camp, 2018; Rosengren and Colebatch, 2018) and can be thought of as a short latency fragment of the vestibulo-collic reflex (Forbes et al., 2018). As illustrated in Figure 1, the VEMP is thought to correspond to a reflex arc involving the vestibular periphery including the VIII cranial nerve, vestibular nuclei, the medial vestibulospinal tract, the XI cranial nerve and the SCM. The exact trajectory through vestibular nuclei is subject to ongoing investigation (Forbes et al., 2013).

The VEMP is a large potential with a characteristic peak (p1) and trough (n1). It has been described as a superposition of motor unit action potentials (Wit and Kingma, 2006; Lütkenhöner, 2019). Wei et al. (2013) measured tuning curves with nine frequencies between 125 and 4,000 Hz, and suggested that VEMPs could be modelled as linear summation of two mass spring systems, with resonance frequencies at approximately 300 Hz and 1,000 Hz. Rosengren et al. (2016) used concentric needle electrodes to measure VEMPs in human participants. Their suggestion was that p1 behaves as a travelling wave, whereas n1 is a combination of a trailing dipole following the propagating inhibition, a standing wave generated when the inhibition reaches the end of the muscle, and a small rebound in firing following inhibition.

Despite VEMP having been well-characterised in animal models including mouse, rat, guinea pig, squirrel monkey, cat and chinchilla (Corneil and Camp, 2018), protocols have varied between experiments and there is no established pre-clinical model for VEMP in rodents or other non-human animals (Raciti et al., 2023). Clinical protocols for VEMP in humans have been established, yet these have come under scrutiny following reports of sudden bilateral hearing loss following testing with the very high air-conducted (AC) sound levels necessary to evoke a VEMP response (Mattingly et al., 2015; Portnuff et al., 2017; Asakura and Kamogashira, 2021). The current study assessed VEMP using a procedure designed to have higher sensitivity than standard clinical procedure (e.g., as per British Society of Audiology, 2012). This used a custom head bar with biofeedback to control neck tension. It also exclusively used body-conducted (BC) stimuli, since these have been found to elicit a VEMP response at appreciably lower levels than are necessary when using AC stimuli (McNerney and Burkard, 2011). As well as eliminating concerns over safe sound levels, BC stimuli enabled collection of substantially more data during a single session than if AC stimuli had been used. This in turn enabled statistical analysis using linear mixed effects regression modelling, which increased statistical power compared to the ANOVA tests typically used, and thereby increased sensitivity.

The normative data reported here were collected as the precursor to a pre-registered study (Gattie et al., 2021) which required high sensitivity in its assessment of a hypothesised difference between groups that do and do not have persistent developmental stuttering. The pre-registration for that study (Gattie et al., 2019) had predicted that VEMP would either have a shortened or prolonged latency, or would have a smaller amplitude, in a stutter group (*n* = 15) compared to non-stutter controls (*n* = 15) paired on age and sex, with the sample sizes calculated from a power analysis using data from a pilot study. People who stutter are difficult to recruit (Gattie et al., 2024) and are a vulnerable group having various subtle differences from people who do not stutter in auditory measures including hyperacusis (see review in Gattie et al., 2021). It was therefore decided to conduct normative testing using the novel VEMP procedure prior to recruitment of people who stutter. A convenience sample of undergraduate students was recruited, with equal numbers of women and men such there would be a principled basis for group comparison using the same statistical analyses as intended for the stutter study. The expectation was of null sex difference on VEMP latency and amplitude, consistent with the studies reviewed in section 3.4 of the current report. For this reason a power analysis was not carried out and the study was not pre-registered.

## 2 Materials and methods

### 2.1 Participants

Participants were 48 students. Equal numbers of women and men participated. Women were aged between 16.6 and 21.1 years (mean 19.22, SD 0.70) and men were aged between 18.6 and 20.7 years (mean 19.72, SD 0.57). Women and men self-identified with the distinguishing characteristic being sex as defined biologically according to the SAGER guidelines (Heidari et al., 2016). Otoscopy, tympanometry and pure tone audiometry were performed following British Society of Audiology guidelines for all participants, with results within the normal range (British Society of Audiology, 2022, 2018, 2014). All participants gave written informed consent according to the Declaration of Helsinki. The University of Manchester Ethics Committee approved the study.

### 2.2 Experimental arrangement

#### 2.2.1 Stimuli

Stimuli were 500 Hz tone bursts with rectangular windowing, created by an Interacoustics Eclipse EP25 system (Interacoustics AS, Assens, Denmark). The frequency of 500 Hz has been shown as optimal for VEMP testing (Rosengren et al., 2010; Papathanasiou et al., 2014). BC 0–1-0 tone bursts with a rise/fall time of zero and a plateau of 2 ms were used, for an overall characteristic between a tone burst and a click (Laukli and Burkard, 2015).

This report presents BC stimulus levels using hearing level (HL) equivalent units, rather than the force level units that are typically used to report vibratory stimuli. Doing so facilitates comparison with studies reviewed in section 3, which report VEMP inconsistently using scales such as dB SPL, dB nHL and dB peSPL. It should be emphasised that the dB HL scale used in this report is dissimilar to the HL scales used for pure tone audiometry (e.g., dB HL re: ANSI S3.6–1996 or dB HL re: ISO 389-1:2017) and is also dissimilar from the dB nHL and eHL scales used in ABR testing (NHSP Clinical Group, 2013). This was unavoidable due to absence of a reference equivalent threshold for the transducer and stimulus chosen, and reflects an ongoing lack of standardisation in calibration procedures for acoustic transients (Burkard, 2006; Lightfoot et al., 2007; Laukli and Burkard, 2015).

BC stimuli were delivered to the mastoid bone behind the right ear at a rate of 5.1 every second, using a B81 bone conductor (Radioear, MN, United States). The procedure for calibration of the bone conductor used a Model 4930 artificial mastoid and 2250 Investigator (Brüel and Kjaer, Naerum, Denmark), and Agilent 54621A 2-Channel Oscilloscope (Keysight, CA, United States). The artificial mastoid had a reference equivalent threshold force level re 1 μN of 40.2 dB for 500 Hz. This reference equivalence was used with a correction factor, provided by Interacoustics, of 69.5 dB for peSPL to nHL conversion of a 2–2-2 500 Hz tone burst; however, and as already noted, this was applied to a 0–1-0 500 Hz tone burst. Based on normative data (e.g., Beattie and Rochverger, 2001; Gorga et al., 2006), inaccuracy introduced by doing so is unlikely to be greater than 5 dB. Any inaccuracy in the threshold reference will in any event not have affected between group comparisons, since both groups were equally affected. Examination of the output using the artificial mastoid and an oscilloscope showed clipping of the trace when amplitudes exceeded 40 dB HL. Stimuli were accordingly set so they would not exceed 40 dB HL.

#### 2.2.2 VEMP recording

VEMP was assessed using the Interacoustics Eclipse. Skin was prepared with NuPrep^®^ (Weaver and Company, CO, United States) prior to electrode attachment with Ten20^®^ conductive paste (Weaver and Company, CO, United States). Non-metallic silver chloride disposable electrodes were used (type M0835, Biosense Medical, Essex, United Kingdom). Electrode impedance was below 3 kΩ. The montage included an active electrode on the right hand side SCM, and reference and ground electrodes on the upper sternum and nasion, respectively.

The Eclipse was used to amplify, record and filter the electromyography (EMG) signal using the Interacoustics research license. A digital FIR filter of 102nd order was set to low pass at 1500 Hz, and an analog Butterworth filter of 1st order at 6 dB per octave was set to high pass at 10 Hz. Sampling by the Eclipse was at the highest resolution available for VEMP protocols, which was 3 kHz. The 3 kHz sampling rate meant that measurements of VEMP p1–n1 latency were grouped into steps of 0.33 ms.

### 2.3 Procedure

**Figure 2:**
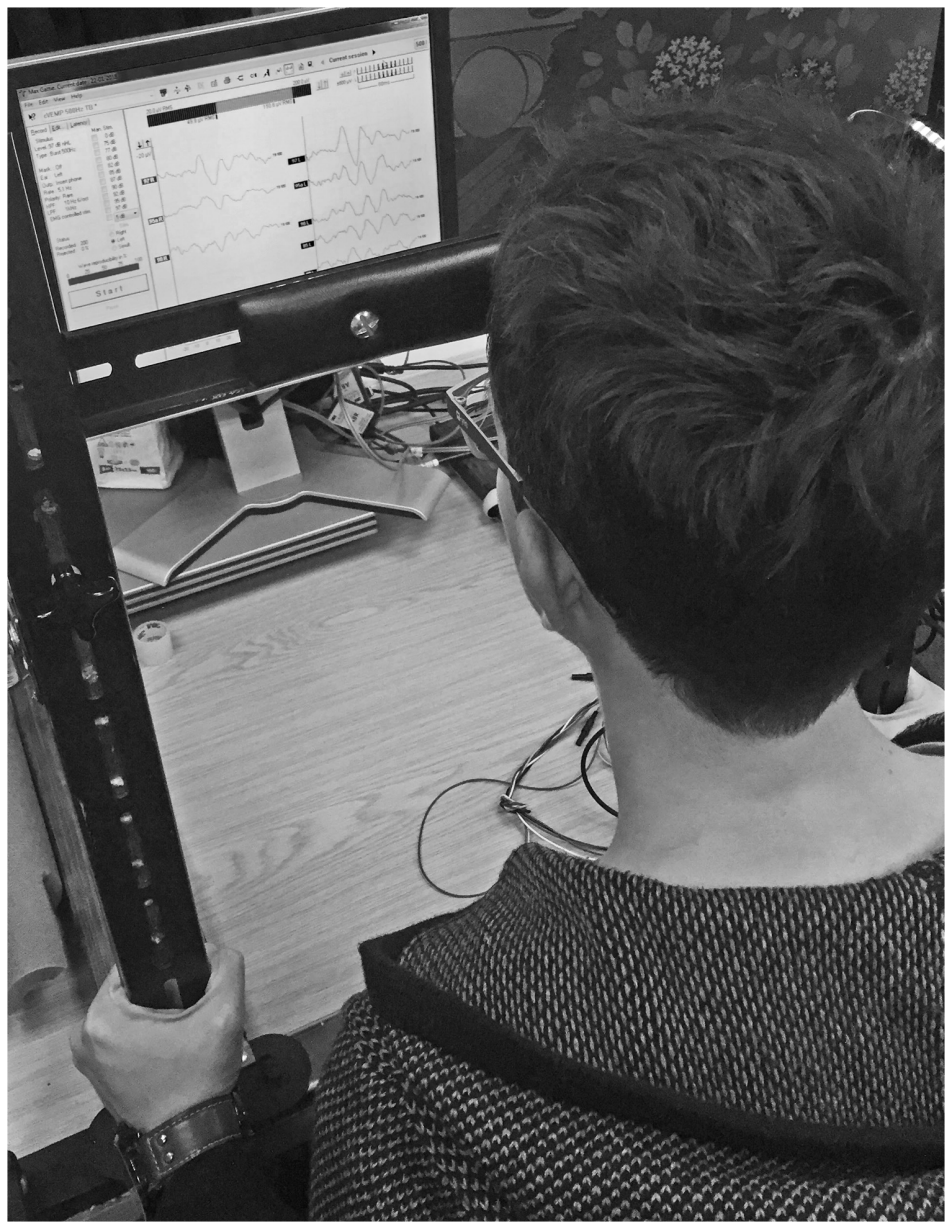
Participants were asked to push against a padded bar with their foreheads, maintaining tension in the sternocleidomastoid tension as close as possible to 50 µV root mean square throughout testing. The Eclipse clinical software provided biofeedback helping participants to monitor sternocleidomastoid tension.

Participants sat with their foreheads resting against a padded bar. The apparatus was specifically constructed for the experiment (Figure 2). Participants were asked to push their heads on the padded bar and try to maintain an EMG biofeedback target as close as possible to 50 μV root mean square (RMS). If background EMG fell lower than 50 μV RMS, stimuli stopped playing and participants were asked to push harder. Participants were instructed not to push harder than necessary to keep the stimuli playing, and would rarely attempt to do so. The importance of maintaining a constant background EMG was explained. Background EMG was monitored by the experimenter throughout testing.

Recordings followed the procedure recommended by Interacoustics for VEMPs. Epochs with peak or trough amplitudes having a magnitude larger than ±800 μV were rejected. The Eclipse software compensated for rejected epochs, so that for every stimulus level tested an averaged response to exactly 300 epochs was recorded. These averages of 300 epochs will henceforth be referred to as “sequences.” Recording of the initial sequence was at a stimulus level of 40 dB HL, and further sequences were recorded with the stimulus level decreased in steps of 2 dB until either a presentation at 34 dB HL, or until the averaged VEMP trace which summarised the sequence was comparable to background noise, whichever came soonest. The experimenter compared the averaged VEMP trace to background noise using the EP25 clinical software. A second series of recordings started at 39 dB HL, with stimulus level decreased in 2 dB steps until either a presentation at 35 dB HL or until the averaged VEMP trace which summarised the sequence was comparable to background noise, whichever came soonest. The plan for data collection was described to participants, who were able to watch their averaged VEMP traces calculated in real time by the EP25 software. If participants were willing (e.g., in the event of no subsequent appointment) and they had shown a response at 34 dB HL, further sequences at stimulus levels below 34 dB HL were recorded. To complete the session, a repeat recording was made using the maximum 40 dB HL stimulus level.

### 2.4 Data processing

Custom scripts were written in MATLAB 2019a (The MathWorks, Inc., Natick, MA) and used to process raw data. Normalisation was carried out for each participant by transforming response amplitudes into a dimensionless ratio. This worked by extracting a pre-stimulus interval of 18 ms from a mean of the EMG waveforms from the first six sequences of 300 presentations recorded for each participant (i.e., the extract was a pre-stimulus mean of the first 1800 presentations recorded). A root mean square (RMS) based on this pre-stimulus mean was then assigned as a background EMG tension for each participant. Finally, each sequence was normalised by dividing it by the background EMG tension for its participant.

The normalisation procedure described was in principle not necessary, since use of the head bar limited variation in background EMG tension according to the 50 μV biofeedback target. However, the normalisation increased accuracy by adjusting for any small per participant variation in EMG tension. Normalisation used the maximum 1,800 presentations available for every participant, and thus minimised random noise in the pre-stimulus RMS background EMG tension. This procedure was considered preferable to alternatives such as per sequence normalisation based on pre-stimulus RMS for each sequence of 300 presentations. Per sequence normalisation could have introduced noise because random fluctuation in pre-stimulus RMS per sequence (i.e., random in addition to any genuine change in sternocleidomastoid tension) would have randomly affected VEMP amplitudes on a per sequence basis, and thus affected within participant comparisons. Comparison of data between participants used linear mixed-effects regression analysis, and this in turn relied upon accurately measuring VEMP amplitude growth with stimulus level for each participant. Thus, preserving within participant comparisons as accurately as possible was optimal for linear mixed-effects regression analysis. The normalisation procedures used in this study preserved within participant comparisons with the same accuracy as that available from the raw data.

**Figure 3:**
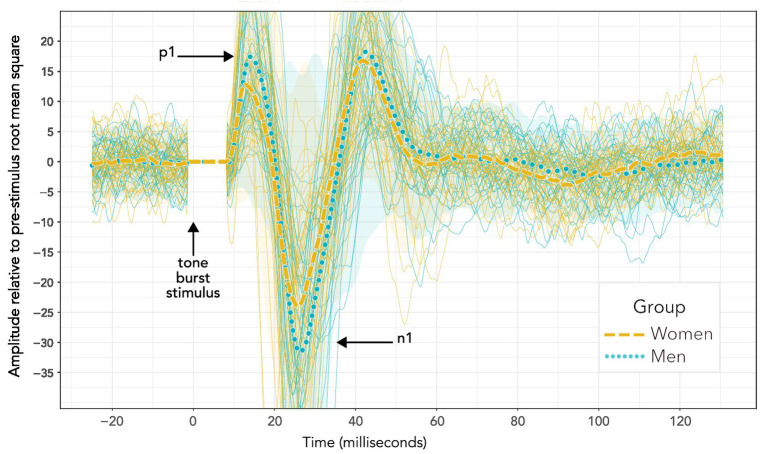
VEMP grand averages recorded following 500 Hz tone bursts presented using bone conduction at 40 dB HL. Distinct sequences for individuals within each group are also shown (more lightly drawn traces than the grand averages) along with 95% confidence intervals (shaded areas). Visual inspection of the waveforms suggests a difference in p1–n1 amplitude, but not p1–n1 latency. However, such an interpretation is misleading. Firstly, these data do not enable a valid statistical comparison, since there were uneven presentation counts across women and men. Secondly, averaging the data to enable statistical comparison shows a significant group difference in p1–n1 latency but not p1–n1 amplitude. Statistical analyses are reported in section 3, and discussed in section 4.

Figure 3 compares VEMP grand averages for women and men at the maximum 40 dB HL stimulus level. Peaks per sequence per participant were identified with the “findpeaks” algorithm in the MATLAB Signal Processing Toolbox. Troughs were identified by applying “findpeaks” to inverted waveforms. To begin with, peaks and troughs were identified for the initial 40 dB HL sequence per participant. This was done by first identifying all of the troughs in the 40 dB HL sequence, and then identifying as n1 the most prominent trough between 15 and 37 ms (with prominence defined as per the “findpeaks” algorithm). Following that, peaks were identified across the entire 40 dB HL trace. Peaks were discarded if they occurred earlier than 5 ms or later than n1. The peaks surviving this process were ranked. Firstly, the three peaks with greatest prominence were awarded 5, 4 and 3 points in order of prominence. Secondly, those three most prominent peaks were weighted according to their prominence relative to the most prominent peak: 3 points for greater than or equal to two thirds; 2 points for greater than one third and less than two thirds; and 1 point otherwise. Thirdly, the five peaks having latencies with the smallest time differences from n1 were given points from 5 to 1 in a hierarchy where higher points were awarded for a smaller time difference. Finally, all of the points were added together. The peak which had the greatest number of points was identified as p1. In the event of a tie, the chosen peak had the smallest time difference from n1.

For other stimulus levels, peaks and troughs were identified using a similar process to that just described for the initial 40 dB HL sequence. A difference was that the trough from the initial 40 dB HL sequence was used as an anchor for trough detection for remaining sequences on a per participant basis. A null identification of peaks and trough occurred (no result was returned from the script) if the p1–n1 amplitude was less than 1.65 times the pre-stimulus RMS for the sequence of 300 repetitions being evaluated.

The script was verified through visual inspection of waveforms for the entire data set collected. Creation of the procedure was via an iterative process, with adjustments made to some of the parameters which have been described prior to re-running the script. Visual inspection showed the final script identifying peaks and troughs accurately. Identification made by the script was final – data points were not removed or adjusted manually.

Data were converted into a response level (RL) scale by taking the logarithm of p1–n1 amplitude as in Equation 1:


(1) $$p1n1amp\;\left(\mathrm{dB}\;RL\right)=20\times \log_{10}\left(\begin{array}{c} p1n1amp\;\left(\mu V\right)\\ \bar{pre-\mathrm{stimulus}\;RMS\;\left(\mu V\right)} \end{array}\right)-20$$

Zero dB RL corresponds to a projected VEMP threshold (this projection is unlike the VEMP thresholds identified in clinical procedure). The transformation is similar to that for the dB SPL scale popular for sound pressure levels, and its frequency-adjusted HL variant.

**Figure 4:**
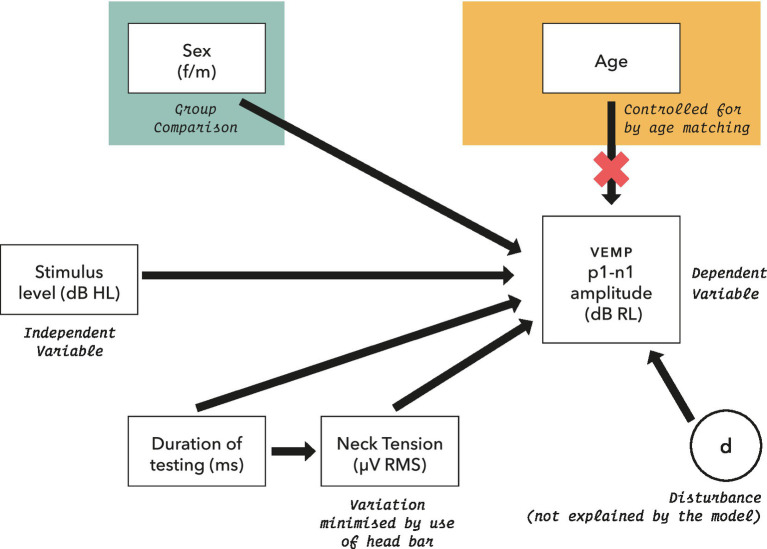
Initial model for statistical analysis of VEMP p1–n1 amplitude measurements. Potential confounders were reviewed in section 2.5, and were assessed as part of the model where possible.

The initial statistical model for VEMP p1–n1 amplitude is shown in Figure 4. It includes potential confounders, as were described in full in Gattie et al. (2021).

Linear mixed-effects regression modelling has been shown to entail a trade-off. There is an increased possibility of type I error when data from all participants are assigned the same slope but can have varying intercepts, versus lower statistical power when both slope and intercept can vary per participant (Barr et al., 2013; Matuschek et al., 2017). Use of a fixed slope model was supported by the precursor to this study (Gattie et al., 2021), which assessed fixed and varying slope linear mixed-effects regression models (Winter, 2019) for VEMP p1–n1 amplitudes with participants who did and did not stutter, and found that a fixed slopes model was most appropriate. Accordingly, a fixed slope model was used in analysis of the current study, with a form as in Equation 2:


(2) $$\mathrm{VEMP}\;p1–n1\;\mathrm{amplitude}=\beta_{0_{j}}+\beta_{1}\times \mathrm{sex}+E$$

VEMP p1–n1 amplitude was conditioned on whether participants were female or male, with ß_0_ as intercept (varies with participant, j) and ß_1_ as a fixed slope of increase in VEMP p1–n1 amplitude with stimulus level. Statistical analysis was conducted with the lme4 package (Bates et al., 2015) in R (R Core Team, 2020). Effect size (Cohen’s d) was calculated from mixed model t statistics with the EMAtools package for R, version 0.1.3 (R Core Team, 2020). Conditional R^2^ was calculated according to Nakagawa et al. (2017) using the MuMIn package, version 1.43.17 (R Core Team, 2020).

### 2.5 Review of prior studies

In order to compare results of the current study with those of prior studies, a literature search was carried out using the query:

VEMP and (sex or male or female or men or women).

Search of titles and abstracts was carried out using OVID database search (Wolters Kluwer N.V., Amsterdam, Netherlands), with the following repositories selected:

All Evidence-Based Medicine (EBM) Reviews – Cochrane, ACP, HTA, DARE, CCA, CCTR, CMR, HTA, NHSEED.
EMBASE.
Health and Psychosocial Instruments.
EBM Reviews – Cochrane Database of Systematic Reviews.
APA PsycInfo.
APA PsycArticles Full Text.
Health Management Information Consortium.
OVID Medline.

Articles were restricted to those in English language only. There were no restrictions for grey literature (e.g., Ph.D. dissertations, pre-prints, conference proceedings). Abstracts were reviewed by hand. Inclusion criteria were that studies had to include VEMP assessment of patients without co-morbidities, and had to report a comparison of p1 and n1 amplitude and latency between women and men. Review focused on data which would enable an appraisal of statistical power relative to the current study. Criteria for comparison between studies included number of partipants, age and sex matching, type and quantity of stimuli used and results of the sex comparison.

## 3 Results

### 3.1 VEMP p1–n1 latency

**Figure 5:**
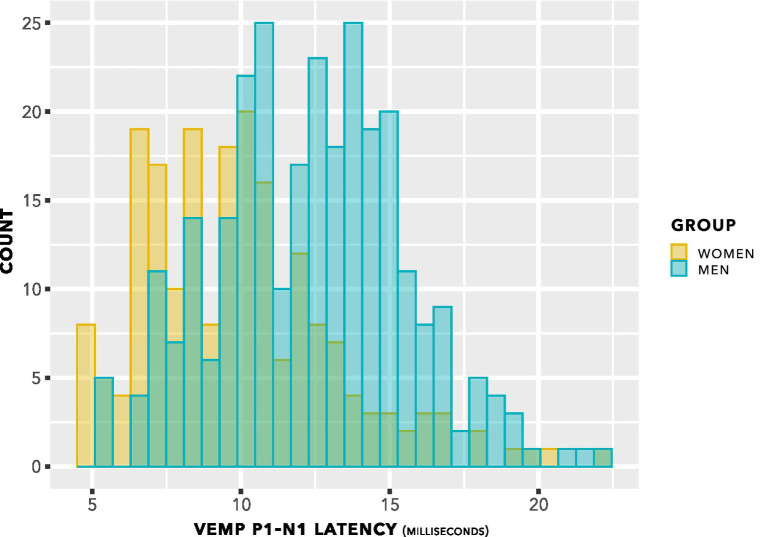
VEMP p1–n1 latency histogram. Indication is of approximately normal distributions for the female and male groups, with some suggestion of a difference in means of the distributions.

The histogram in Figure 5 shows counts of VEMP p1–n1 latency measurements sorted into female and male participants. The histogram does not show detail of participant or stimulus level. Inclusion of repeated measurements in the histogram means that it is not appropriate for statistical comparisons. However, the presentation count was approximately equal per participant, and was carried out over approximately the same stimulus range. Therefore the histogram gives an indication of distribution for each group. Both the female and male groups appear to have approximately normal distributions. It is apparent that slightly more data have been collected from men than from women. There is a suggestion of difference between the means of the group distributions.

**Figure 6:**
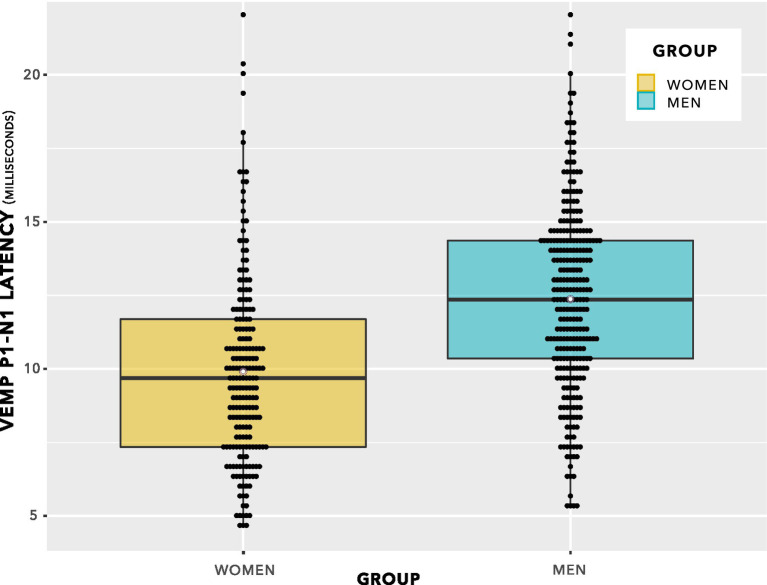
VEMP p1–n1 latencies. The even spacing on the ordinate was due to the 3 kHz sampling rate. VEMP p1–n1 latencies are grouped in steps of 0.33 ms, which was the maximum resolution of the Eclipse when using VEMP protocols. To provide an indication of when several data points were recorded at the same VEMP p1–n1 latency, data points have been plotted using the beeswarm feature in ggplot (R Core Team, 2020). This feature has introduced variation along the abscissa corresponding to the quantity of data present. Mean values for each group are shown as white data points. The box plot suggests there will be a group difference in VEMP p1–n1 latency, however it contains repeated measurements per participant and as such does not entirely reflect data appropriate for use in statistical analysis. Statistical analyses are reported in section 3, and discussed in section 4.

The data from Figure 5 are shown in a box plot in Figure 6. There appears to be a group difference.

Statistical evaluation was carried out by linear mixed-effects modelling. First, the effect of stimulus level on VEMP p1–n1 latency was appraised. The following models were compared:

model_null: latency ∼ 1 + group + (1| participant).
model_diff: latency ∼ stimulus + group + (1| participant).

**Figure 7:**
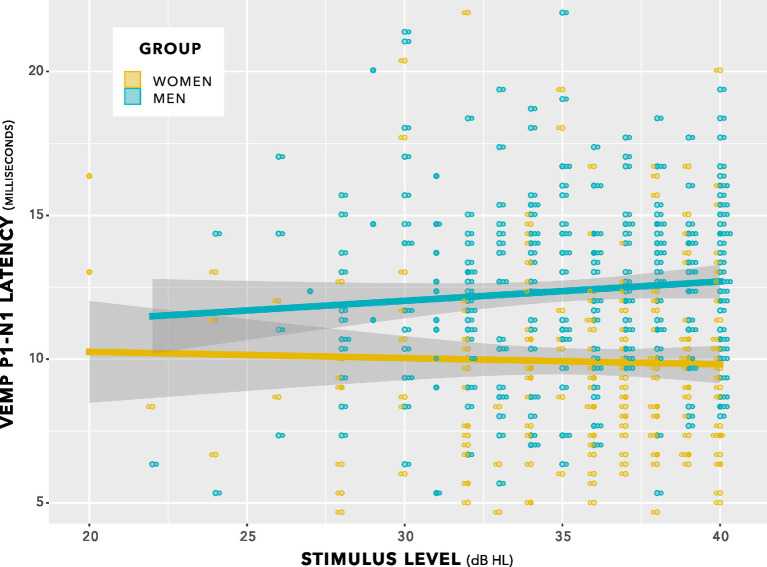
VEMP p1–n1 latencies versus stimulus level for women and men. There was no interaction. Sampling resolution of the Eclipse meant that data points were grouped in steps of 0.33 ms (see note at Figure 5). To provide an indication of when data from both women and men were recorded at the same stimulus level and VEMP p1–n1 latency, data points have been plotted using the beeswarm feature in ggplot (R Core Team, 2020). This feature introduced variation along the abscissa corresponding to the quantity of data present. The variation does not reflect stimulus level used. Stimulus level was always an integer multiple of 1 dB.

This comparison showed no significant difference between models (chi squared (1) 0.06, *p* = 0.80). The indication was of no effect of stimulus level on p1–n1 latency. This is illustrated in Figure 7. Further comparisons indicated no effect of duration of testing or of neck tension.

Next, *p*-values were generated by likelihood ratio comparisons between the following models.

model_null: latency ∼ 1 + (1| participant).
model_diff: latency ∼ 1 + group + (1| participant).

There was a significant difference between models (chi squared (1) 9.6, *p* = 0.002).

The data showed that VEMP p1–n1 latency was shorter for women than for men by 2.43 ms (95% CI [−0.93, −3.92], chi squared (1) 9.6, *p* = 0.0020). Data used in this analysis are available in the Supplementary material.

An alternative method for statistical analysis would be to average repeated measures per participant, enabling statistical comparison using a Welch’s unequal variances t-test. This alternative analysis forms the basis for the power calculations conducted in section 4, and so is reported here. The finding from the t-test is that p1–n1 latency was 2.4 ms shorter in women than in men. This is statistically significant at *p* = 0.0026 (95% CI -0.89, −3.96; t stat −3.18, df 46, mean (women) 10.0, variance 7.5; mean (women) 10.0; mean (men) 12.4; variance 6.4). It compares with *p* = 0.0020 for the linear mixed-effects modelling comparison, as described immediately above.

### 3.2 Absolute p1 and n1 VEMP latencies

Absolute p1 and n1 VEMP latencies were evaluated, using linear mixed-effects modelling similar to that already described for p1–n1 peak to trough latency. These analyses are made available for readers wishing to compare results from this study with the prior studies (see Table 1) which have reported absolute p1 and n1 VEMP latencies. These additional analyses are presented for exploratory purposes only. Analyses of absolute VEMP p1 and n1 latencies were not pre-registered, multiple corrections have not been made enabling such additional analyses, and results from the additional analyses are not a focus of discussion in this report.

**Table 1:** Studies comparing cervical vestibular-evoked myogentic potential (VEMP) latencies in women (F) and men (M).

Authors	Participants	Stimuli	Presentations	Result
Welgampola and Colebatch (2001)	34F, 36 M, aged 25–85 years. No age breakdown provided according to sex.	AC 100 dB nHL clicks	256 clicks delivered per ear 512 total presentations	Amplitude: Women had larger p1–n1 amplitude (*p* = 0.02 after excluding participants older than 80 years). Latency: A sex-specific breakdown of latency data was not reported.
Brantberg and Fransson (2001)	11F, 12 M, aged 22–42 years. No age breakdown provided according to sex.	AC 100 dB nHL clicks.	128 clicks per sequence, 768 total presentations. Six conditions (monaural right/left and four binaural presentations at different rates). Two sequences per condition.	Amplitude: No significant sex differences. Latency: p1 was shorter in women than men by 0.74 ms (*p* < 0.001) for binaural clicks, and 0.89 ms (*p* < 0.05) for monaural clicks.
Ochi and Ohashi (2003)	28 M, 32F, aged 20–77 years (mean 40.6, SD 16.8), age matched in aggregate.	AC clicks presented in 5 dB increments at 105 dB peSPL and lower.	Usually (at least) 50 stimuli per sequence.	Amplitude: No significant sex difference. Latency: A sex-specific breakdown of latency data was not reported.
Akin et al. (2003)	9F, 10 M, aged 22–51 years. No age breakdown provided according to sex.	AC clicks presented in 5 dB increments between 80 dB HL and 100 dB HL.	No detail of number of stimuli per sequence. Amplitudes and latencies calculated from mean of three recordings per click level.	Amplitude: No significant sex difference Latency: No significant sex difference
Basta et al. (2005, 2007)	38F (43.7 years ±12.6), 26 M (49.6 years ±14.6), age range 20–76 years.	AC and BC 500 Hz tone bursts, 7 ms duration.	No detail of number of stimuli per sequence. Two sequences per participant.	Amplitude: No significant sex difference (Basta et al., 2007) Latency: no significant sex difference (Basta et al., 2005)
Brantberg et al. (2007)	625F, 375 M, aged 7–91 years. Study analyses effects of sex and age differences.	AC 2–2-2500 Hz tone burst, C-weighted max peak value of 129 dB SPL.	Each sequence contained 64 tone bursts. Three sequences per participant. 192 total presentations.	Amplitude: no significant difference in p1–n1 amplitude. Latency: No significant sex difference in n1 latency. Age-sex interaction in p1 latency for women (p < 0.05), i.e., p1 latency in women not as prolonged with higher age as for men.
Lee et al. (2008)	48F, 49 M, aged 12–77 years. F mean age 42 ± 17y, M mean age 43 ± 18y. Age stratified groups, but no detail of sex ratio within age stratified groups.	AC clicks at 95 dB HL.	Detail of recording duration not apparent.	Amplitude: Women had significantly larger p1–n1 amplitude (*p* = 0.04) Latency: n1 occurred 1.4 ms earlier in women than men (p < 0.001). Not quite reaching significance (*p* = 0.06) p1 was 1.0 ms earlier than in men.
Tourtillott et al. (2010)	14F, 15 M, age stratified into groups 23–30y, 65–71y and 75–84y.	AC 500 Hz 2–1-2 tone burst at a maximum of 95 dB HL.	150 presentations per sequence. Just the 95 dB HL sequence used for amplitude/latency comparisons, further sequences used for clinical threshold search.	Amplitude: No significant sex difference Latency: No significant sex difference
Carnaúba et al. (2011)	40F, 40 M, “young individuals” (no detail of age matching)	AC 90 dB HL 500 Hz tone bursts.	No detail of number of stimuli in sequence. Two sequences per side per participant (i.e., four total)	Amplitude: No significant sex difference Latency: No significant sex difference
de Oliveira Barreto et al. (2011)	40F, 38 M, 18–31 years old, mean age 21.3 years, SD 2.29 years	AC 4–2-4 tone bursts at 250, 500, 1,000, and 2000 Hz	200 presentations per frequency tested. 800 presentations total.	Amplitude: 250 Hz and 500 Hz no significant sex difference. 1,000 and 2000 Hz, p1–n1 interpeak amplitude smaller in men than women. Latency: 250 Hz, 500 Hz no significant sex difference. 1,000 Hz p1–n1 interpeak latency shorter in women than men (*p* = 0.002), 2000 Hz n1 shorter in women than men (p = 0.02).
Rosengren et al. (2011)	28F, 33 M, age stratified in decades from 20 to 80 years old.	AC clicks at 135 dB peak SPL, and AC 2 ms 500 Hz tone bursts at 132 dB peak SPL. BC 500 Hz tone bursts at 136 dB peak FL. Also used mini-shaker pulses (4 ms rise time, peak amplitude of 131 dB FL) and taps via reflex tendon hammer.	256 presentations of each stimulus, except taps, which had 40 presentations.	Amplitude: No significant sex difference Latency: No significant sex difference
Gazioglu and Boz (2012)	24F, 11 M, 21–54 years, age matched in aggregate (mean age 33.6 ± 9.7).	AC clicks at 125 dB nHL	Two sequences of 128 presentations.	Amplitude: No significant sex difference Latency: No significant sex difference:
Khan et al. (2014)	17F, 68 M, age stratified from 7 to 71 years. Mean age 26F, 28 M.	AC click and 500 Hz tone burst, maximum stimulus level 100 dB nHL.	Between 75 and 250 presentations per sequence. 250 total presentations.	Amplitude: No significant sex difference Latency: No significant sex difference
Silva et al. (2016)	15F, 15 M, aged 18–53 years. F mean age 35 y (SD 8y), M mean age 29 y (SD 7 y)	AC 120 dB HL 500 Hz tone bursts	Minimum two sequences of 100 stimuli; ≥ 200 presentations.	Amplitude: Men had larger p1–n1 amplitude in the right ear (*p* = 0.03) but not in the left ear. Latency: No significant sex difference
Li et al. (2015)	101F, 85 M completed testing. Original sample was 250, mean age 72.6 years (SD 12.5), range 26–92y.	AC 125 dB SPL 500 Hz 1–2-1 tone bursts	Detail of recording duration not apparent.	Amplitude: No significant sex difference. Latency: p1 0.39 ms shorter in women (*p* = 0.005), with n1 latency not reported.
Blakley and Wong (2015)	28F, 20 M, overall age range 23–64 years (mean 36y, SD 13.1y).	AC 500 Hz tone bursts at a maximum of 100 dB nHL	200 presentations to each ear at the maximum 100 dB nHL, then descending in 5 dB steps to clinical threshold; ≥ 200 presentations overall.	Amplitude: No significant sex difference Latency: No significant sex difference
Shahnaz and David (2021)	22F, 22 M, aged between 20–29 years (no detail of age matching)	AC 118.5 dB peSPL 500 Hz 2–2-2 tone bursts,	Minimum two sequences of 200 presentation; ≥ 400 presentations overall.	Amplitude: Men had larger p1–n1 amplitude (*p* = 0.04). Latency: No significant sex difference
Patterson et al. (2021)	47F, 38 M, 10–87 years (mean 48.8 ± 21.9).	AC and BC 1–0-1500 Hz tone bursts, 70–75 dB nHL.	100 presentations per sequence.	Amplitude: No sex difference. Latency: Not reported.
Wiener-Vacher et al. (2023)	42 adults, 31F, 11 M (16–61 years, mean 35.5 years, SD 15 years). Also 118 children (2 months to 15 years), 60 boys, 58 girls.	AC and BC, 100 dB HL, 750 Hz 1–1-1 tone bursts.	Three sequences of usually (at least) 25 presentations in children, 50 presentations in adults.	Amplitude: Boys had larger p1–n1 amplitude than girls, men had larger p1–n1 amplitude than women (*p* = 0.03). Latency: No significant sex difference.
Current study	24F, 24 M. F mean age 19.22 years (SD 0.70). M mean age 19.72 years (SD 0.57)	BC 0–1-0500 Hz tone burst.	300 presentations per sequence. Between 6 and 15 sequences per participant (mean 10.5, SD 3.2); Total ≥ 1800 presentations; mean 3,044 presentations.	Amplitude: No significant sex difference. Latency: Women had shorter p1–n1 inter-peak latency by 2.4 ms (*p* = 0.0020).

Stimuli were air-conducted (AC) in all but the current report, which exclusively used body-conducted (BC) stimuli; and Patterson et al. (2021) and Wiener-Vacher et al. (2023), both of which used AC and BC stimuli. Inconsistencies in detail between summaries reflect the level of detail provided in the original reports. See section 2.2 for discussion of how different calibration systems can be compared.

For women, the absolute p1 latency was 15.3 ms [95% CI 14.5, 16.1] and the absolute n1 latency was 25.3 ms [95% CI 24.3, 26.3]. For men, the absolute p1 latency was 14.6 ms [95% CI 14.2, 15.0] and the absolute n1 latency was 27.0 ms [95% CI 26.1, 27.8]. There was no group difference in p1 latency with *p* ≤ 0.05, although a statistically insignificant and uncorrected p1 prolongation of 0.74 ms in women compared to men was observed (*p* = 0.11, chi squared (1) = 2.54, standard error = 0.46). For n1 latency, there was a statistically significant group difference. The n1 trough occurred 1.69 ms earlier in women than in men (*p* = 0.015, chi squared (1) = 5.90, standard error = 0.67). Even with a Bonferonni correction, this group difference would remain statistically significant at *p* ≤ 0.05.

### 3.3 VEMP p1–n1 amplitudes

**Figure 8:**
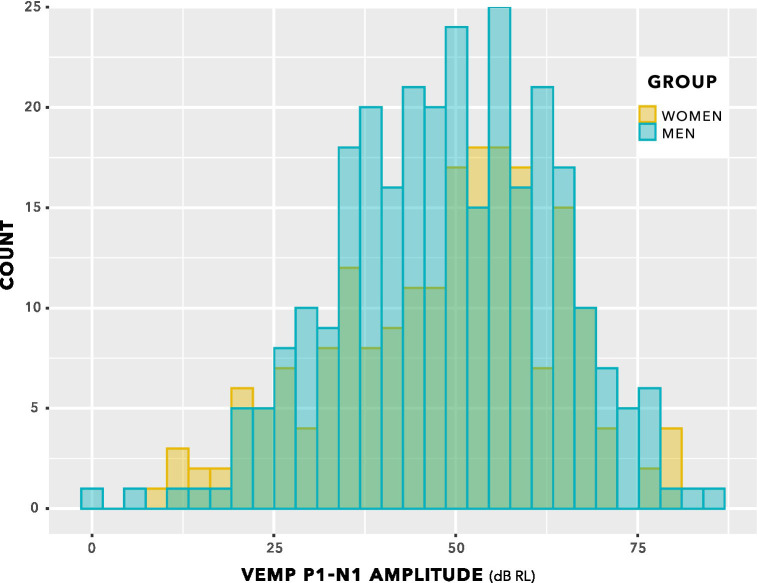
VEMP p1–n1 amplitude histogram. Indication is of approximately normal distributions for the female and male groups. There is no suggestion of a difference in means of the distributions.

Figure 8 shows VEMP p1–n1 amplitudes collected across all participants and all stimulus levels, including repeat measurements. Distribution of data seems approximately normal, with no suggestion of a group difference.

**Figure 9:**
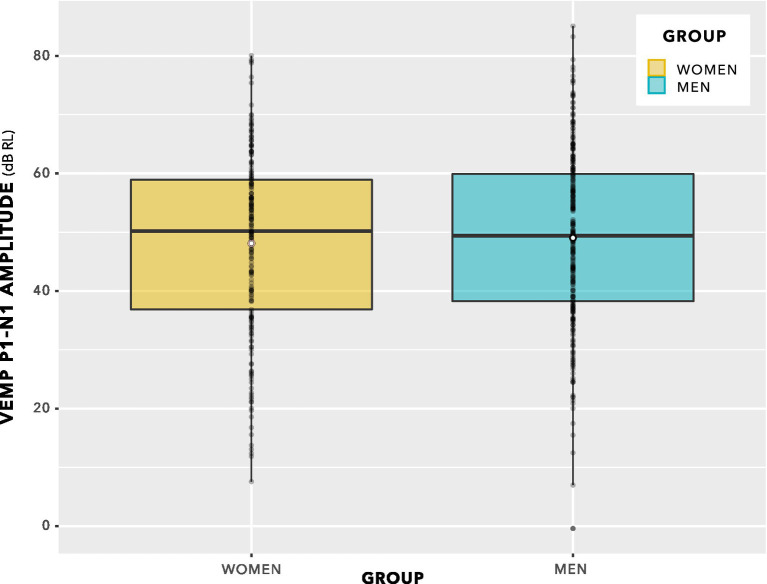
VEMP p1–n1 amplitudes. Since there are repeated measures per participant, this is not a valid statistical comparison. However, there is no indication of a statistically significant group difference. Statistical analysis was via linear mixed-effects regression modelling, and is described in the main text.

The box plot in Figure 9 provides an alternative view of the data in Figure 8. Again, there is no suggestion of group difference.

**Figure 10:**
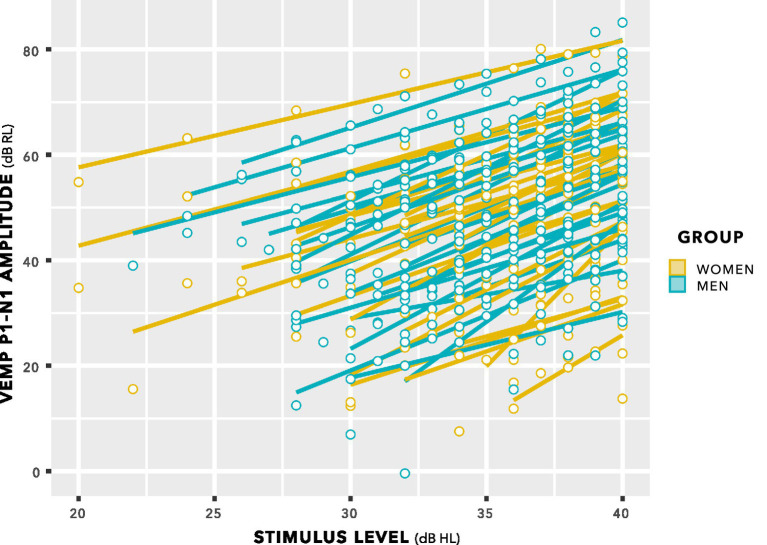
VEMP p1–n1 amplitude versus stimulus level. Individual lines of best fit are per participant. The slopes have a mean value of 1.89 with SD 0.25, supporting use of a fixed slope linear mixed effects model.

The distributions in Figure 9 do not enable a valid statistical comparison, because they include repeated measurements. Statistical comparison was achieved through linear mixed-effects regression modelling, using stimulus level as a predictor, as illustrated in Figure 10. This is a log–log graph, since both the abscissa (stimulus level) and the ordinate (VEMP p1–n1 amplitude) are transformed into logarithmic scales. Thus, a power relationship is apparent between stimulus level and VEMP p1–n1 amplitude. The slopes have a mean value of 1.89 with standard deviation 0.25, supporting use of a fixed slope linear mixed effects model. As in Figures 8, 9, Figure 10 shows no suggestion of group difference.

**Figure 11:**
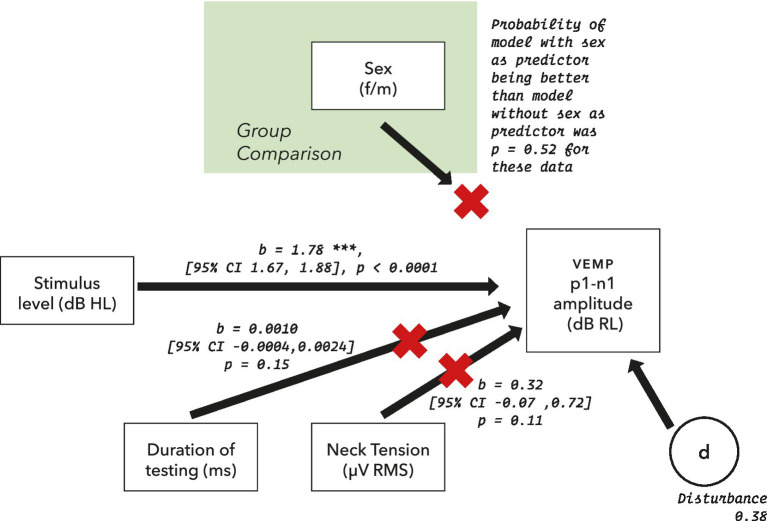
The model for VEMP p1–n1 amplitude described in figure 4, updated following data collection. Only stimulus level predicted VEMP p1–n1 amplitude.

Likelihood ratio comparisons to a nested model in which sex of participant as a predictor was absent was used to calculate *p* values. There was no significant difference between models evaluated (Chi-Squared (1) = 0.41, *p* = 0.52), indicating that VEMP p1–n1 amplitude did not depend on the sex of the participant. Conditional R^2^ was evaluated as 0.92. Overall results are shown in Figure 11.

Both time elapsed and pre-stimulus RMS had *p* > 0.1, and a small effect on VEMP p1–n1 amplitude relative to stimulus level. They were therefore removed from the final model, as indicated in Figure 11. Only stimulus level predicted VEMP p1–n1 amplitude.

### 3.4 Review of prior studies

**Figure 12:**
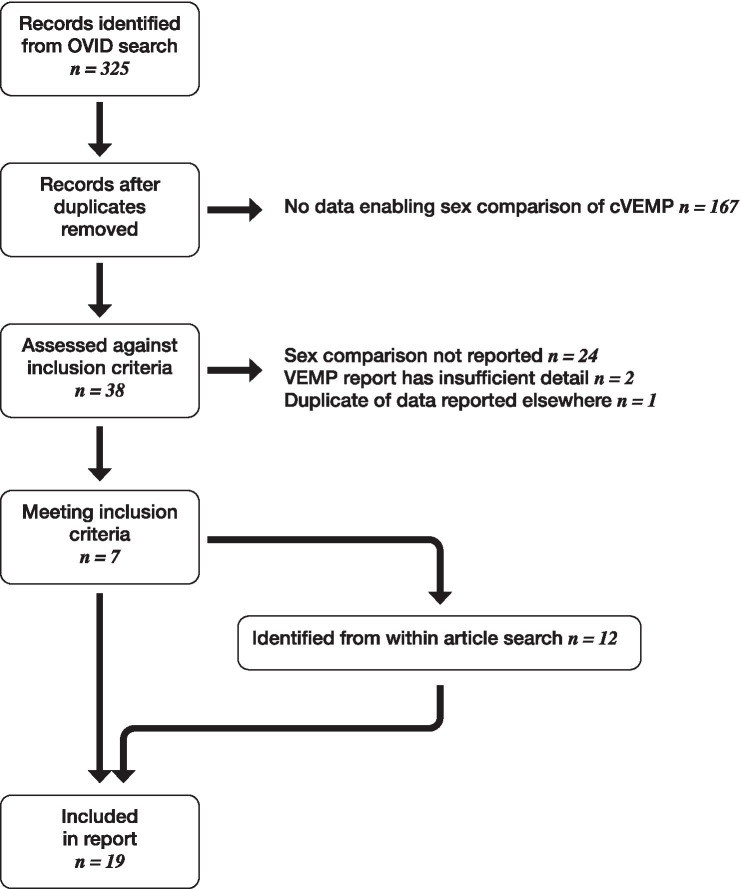
Flow diagram showing literature review process.

A flow diagram summarising the process for literature review is shown in Figure 12.

From 204 studies returned following literature search, 19 were identified meeting inclusion criteria. These are summarised in Table 1, along with results from the current study.

VEMP p1–n1 latency was 2.4 ms shorter for women than men in the current study (95% CI [−0.9, −3.9], *p* = 0.0020). Of 16 prior studies appraising VEMP p1–n1 latency, 11 found no sex difference. Although 5 studies consistently found women with shorter p1 or n1 latencies than men, when the p1–n1 latency difference is considered the direction of sex difference is inconsistent. Three studies found a shorter p1–n1 latency in women than in men (Lee et al., 2008; Brantberg et al., 2007; de Oliveira Barreto et al., 2011, but at 1,000 Hz only) and two studies found a longer p1–n1 latency in women than in men (Brantberg and Fransson, 2001; Li et al., 2015).

VEMP p1–n1 amplitude had no significant difference between women and men in the current study. Of 19 prior studies appraising VEMP p1–n1 amplitude, 13 studies found no sex difference. Of the remainder, three found larger p1–n1 amplitudes in women (Welgampola and Colebatch, 2001; Lee et al., 2008; de Oliveira Barreto et al., 2011, but at 1,000 Hz / 2,000 Hz only), two found larger p1–n1 amplitudes in men (Shahnaz and David, 2021; Wiener-Vacher et al., 2023) and one found larger p1–n1 amplitudes in men but in the right ear only (Silva et al., 2016). Thus, the direction of the difference was inconsistent.

**Figure 13:**
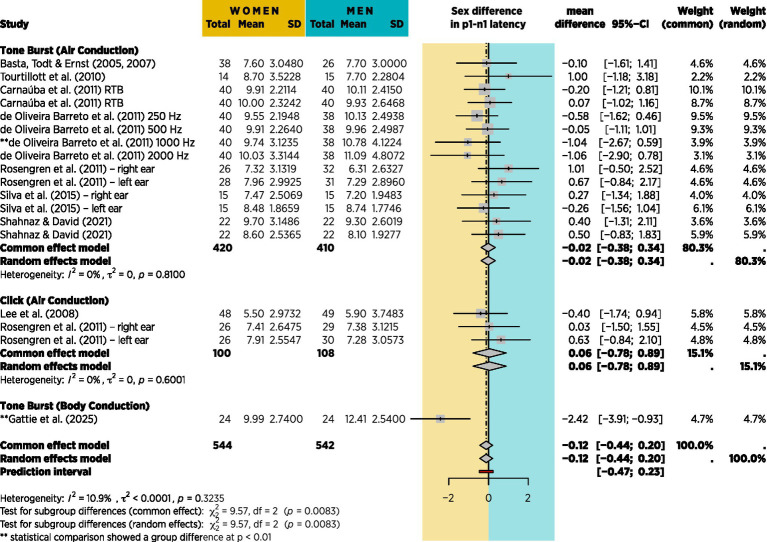
Meta-analysis based on 9 of the 20 studies from table 1 for which mean p1-n1 latency difference with standard deviations could be obtained. No account is taken of stimulus presentation count, however the effect is shown in Figure 14.

Several of the studies identified in Table 1 contained data that would enable a meta-analysis of sex difference in VEMP p1-n1 latency. Efforts were made to retrieve additional data from the authors of other reports. Figure 13 shows the results of meta-analysis based on 9 studies, including the current study, with grouping according to the type of stimulation used. Although 20 studies are identified in Table 1, 10 are missing from the meta-analysis, including 4 of the 6 that found a sex difference in p1-n1 latency. Figure 13 shows 18 sex comparisons in total because when studies included multiple measures (e.g., left and right ears, or clicks and tone bursts) all relevant data were used for meta-analysis, however no adjustment was made for repeated use of the same participants in such studies. Figure 13 is thus based entirely on standard deviations and participant counts, and was conducted according to the procedure described by Shamim et al. (2023) using R (v 4.4.2; R Core Team, 2024) and the packages meta (v8.0–2; Balduzzi et al., 2019).

**Figure 14:**
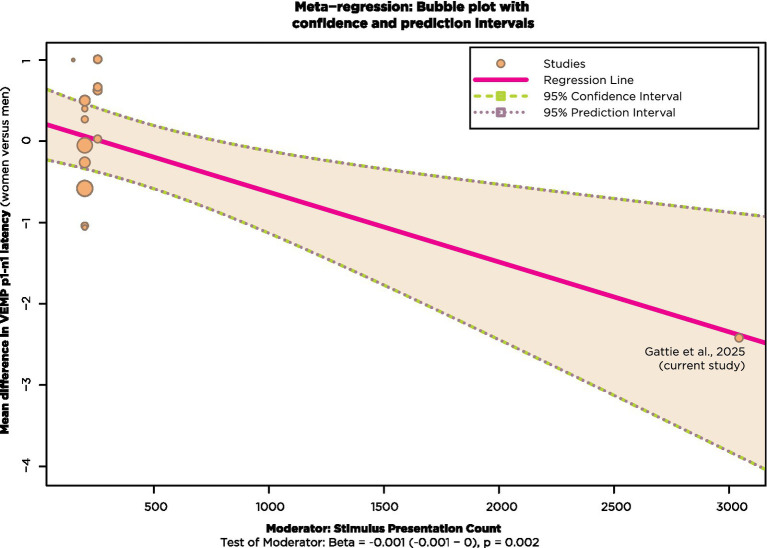
Meta-regression with studies from Figure 13 that also reported stimulus presentation count.

To take account of the differing stimulus presentation counts for the studies in Figure 13, a meta-regression was run with stimulus presentation count as a moderating continuous variable using the R package metafor (v4.8–0; Viechtbauer, 2010). In doing so, it was necessary to exclude the studies of Basta et al. (2005, 2007), Lee et al. (2008), and Carnaúba et al. (2011), since they did not report stimulus presentation count. Results are shown in Figure 14.

Most of the studies in Figure 14 had stimulus presentation counts of approximately 200. These are shown clustered in the top left of Figure 14, with bubble size corresponding to weighting. The effect of stimulus presentation count as a moderator is statistically significant at the *p* = 0.02 level when the current study, shown at the bottom right of Figure 14, is included in the analysis.

## 4 Discussion

### 4.1 Comparison to prior studies

This study found that VEMP p1–n1 latency was shorter in women than men by 2.4 ms (95% CI [−0.9, −3.9], chi squared (1) 9.6, *p* = 0.0020). The VEMP p1–n1 latency difference equates to 21% of the mean 11.4 ms VEMP p1–n1 latency, which was measured across women and men. The latency finding differs from the indication of no difference in prior research (see section 3.4). The study also found that there was no sex difference in VEMP p1–n1 amplitude, which is in line with the indication from prior studies.

There are at least two interpretations of the prior research. In the first, VEMP has no genuine difference in p1–n1 latency or p1–n1 amplitude between women and men. The rationale for this explanation is that in over half of the studies in Table 1 no sex difference in VEMP measures was found, and in the remainder there was no consistent direction of sex difference in either p1–n1 latency or p1–n1 amplitude. The occasional differences established might, for example, have been due to sampling variation, confounders specific to individual studies, or a combination of both. An alternative explanation is that prior studies were underpowered. This second explanation is compatible with aspects of the first explanation (e.g., occasional findings of sex difference due to sampling variation or study-specific confounders), but it adds the detail that a genuine sex difference in VEMP measures could have been missed due to a lack of sensitivity in the tests applied. Three aspects of the current study support this alternative explanation.

Firstly, use of the head bar and biofeedback in the current study provided greater control of SCM tension than the head turn or head raise procedures in prior studies. This has greater importance for measurement of p1–n1 amplitude than for p1–n1 latency, since p1–n1 amplitude is proportional to SCM tension (Ochi et al., 2001). Physiological effects related to SCM tension were discussed in detail in Gattie et al. (2021). The indication from the current study is that there is no sex difference in VEMP p1–n1 amplitude.

Secondly, age matching between sexes was more exact in the current study than in prior studies. Participants in the current study were aged between 18 and 21 years, plus one 16 year old, with a mean age of 19.5 and SD of 0.7. Age matching between sexes in prior studies was appreciably less exact and was sometimes not even reported. Less exact age matching introduces a confounder, since across the lifespan p1–n1 amplitudes are attenuated by a factor of 2 (Welgampola and Colebatch, 2001; Brantberg et al., 2007) and there is variation of between 1 ms and 3 ms in p1 and n1 latencies (Brantberg et al., 2007; Macambira et al., 2017). Thus, the failure to establish a sex difference in some prior studies may be because a genuine sex difference was obscured by an age effect which was not adequately controlled for. Equally, when a sex difference was established, sometimes with a different direction of fit to the current study, or in terms of amplitude rather than latency, the finding may in fact have reflected a variation in age rather than sex between the groups which were compared.

Thirdly, exclusive use of BC stimuli in the current study enabled collection of a far greater amount of data per participant than any other study described in Table 1. The number of stimulus presentations was typically an order of magnitude greater—a mean of 3,044 presentations per participant in the current study, versus between 200 and 300 per participant in prior studies. The effect of collecting a greater number of samples of VEMP responses per participant will have been to decrease sampling variation in the within participant measure. Reduction of sampling variation in the within participant measure in turn reduces the chance of reporting an apparently statistically significant, but in actuality spurious, difference in the between group comparison, and increases the chance of reporting a genuine group difference.

The prior studies in Table 1 were typically not designed for appraisal of sex difference in VEMP measures and, from the considerations just described, may have been underpowered to do so. This possibility could be appraised with a power analysis based on data from the current study. There are serious caveats when interpreting *post hoc* power analyses (Hoenig and Heisey, 2001; Lakens and Evers, 2014). These are compounded in the current study by the difficulty of estimating power in linear mixed-effects regression modelling (Kumle et al., 2021). A standard solution to the latter difficulty is to implement Monte Carlo simulation, however even with stock routines (Green and MacLeod, 2016) the programming overhead would have been beyond scope for this study. Fortunately, there was a simpler solution. As described in section 3.1, averaging VEMP p1–n1 latency measurements per participant then running a Welch’s unequal variances t-test gave near-identical results to the linear mixed-effects regression analysis. Use of t-tests enables adoption of the power analysis routines described by Cohen (1969). Such analyses are reported in Table 2, with power calculations according to G*Power (Faul et al., 2007). The effects of fewer stimulus presentations, and fewer participants, were both modelled.

**Table 2:** Modelling of the effect of presenting fewer stimuli, and/or testing fewer participants.

	No. of presentations per participant	Total no. of participants	Women		Men		Group difference	95% CI		*p*-value	Power
								Lower	Upper		
			Mean	SD	Mean	SD					
Mixed model	3,044 (on average)	48	–	–	–	–	2.43	0.93	3.92	0.0020	–
*t*-test	3,044 (on average)	48	9.99	2.74	12.41	2.54	2.43	0.89	3.96	0.0026	0.88
	1,500	48	9.96	3.05	12.23	2.50	2.27	0.65	3.89	0.0071	0.79
	1,200	48	9.98	2.92	12.32	2.45	2.34	0.77	3.91	0.0043	0.84
	900	48	9.88	2.86	12.28	2.51	2.40	0.83	3.96	0.0034	0.85
	600	48	10.03	3.24	12.60	2.57	2.57	0.87	4.27	0.0039	0.85
	300	48	10.48	3.55	12.37	2.83	1.89	0.03	3.76	0.0470	0.52
	1,500	44	9.90	3.18	12.48	2.41	2.58	0.86	4.30	0.0043	0.84
	1,500	40	10.03	3.30	12.22	2.36	2.18	0.34	4.03	0.0216	0.65
	1,500	36	10.31	3.27	12.36	2.43	2.05	0.09	4.01	0.0404	0.55
	1,500	32	10.65	3.25	12.32	2.52	1.67	−0.44	3.78	0.1158	0.35
	300	44	10.40	3.68	12.53	2.79	2.13	0.13	4.12	0.0370	0.56
	300	40	10.41	3.74	12.23	2.75	1.82	−0.29	3.93	0.0882	0.40
	300	36	10.69	3.72	12.32	2.86	1.63	−0.62	3.89	0.1499	0.30
	300	32	11.00	3.80	12.42	2.85	1.42	−1.01	3.85	0.2421	0.21

The results presented in section 3.1 used linear mixed-effects modelling, with an average of 3,044 presentations for each of 48 participants (24 women, 24 men). As described in the section 3.1, an analysis with near-identical results is possible using a Welch’s unequal variances *t*-test. For the simulation, *t*-tests were recalculated after reducing either participant count or number of presentations. In all simulations, the data which were collected last were removed prior to reanalysis.

**Figure 15:**
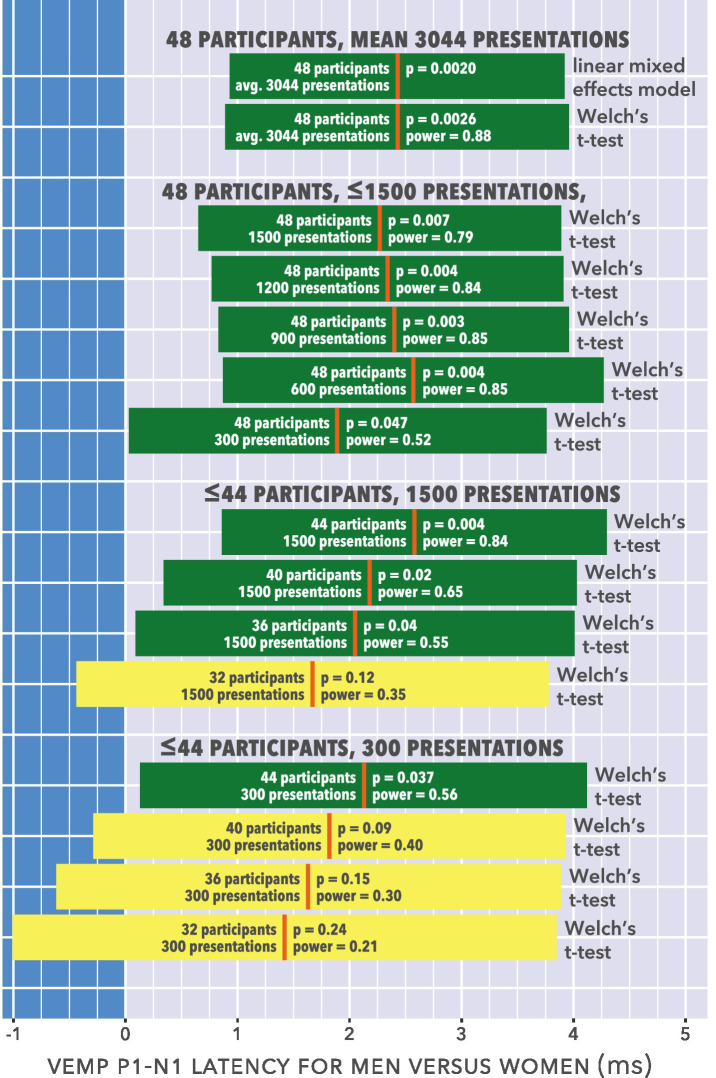
95% confidence intervals for the analyses shown in Table 2. Participant counts were balanced (e.g., 48 participants are 24 women, 24 men). Group differences are shown as prolongation in men’s VEMP p1–n1 latencies compared to those of women. The mean latency difference is shown for each condition as a vertical orange line, with horizontal bars either side showing the 95% confidence interval. When the 95% confidence interval crosses zero, the data analyses in the simulations did not establish a statistically significant group difference. Such analyses are depicted in a lighter colour (yellow), whereas analyses for which a statistically significant group difference could be established are depicted in a darker colour (green).

The analysis is presented graphically in the form of 95% confidence intervals in Figure 15.

Figure 15 suggests that with 300 stimulus presentations per participant, at least 44 participants would be required to establish a sex difference in p1–n1 latency. Five of the studies described in Table 1 met these criteria or were close (Welgampola and Colebatch, 2001; Shahnaz and David, 2021; Rosengren et al., 2011; de Oliveira Barreto et al., 2011; Patterson et al., 2021). One of these found a p1–n1 latency shorter in women than men (de Oliveira Barreto et al., 2011), similar to the current study, however this was for 200 stimulus presentations at 1000 Hz and 2000 Hz only; a sex difference was not found at the 500 Hz frequency used in the current study, nor at 250 Hz. The other four studies did not find a sex difference in p1–n1 latency, however all are difficult to interpret on latency measures. One assessed 34 women and 36 men having an age range of 25–85 years and did not report an age breakdown according to sex (Welgampola and Colebatch, 2001). This introduced the possibility that changes expected to occur in latency due to age (Macambira et al., 2017) were unbalanced with regard to sex, making the study unsuitable for comparison of VEMP latencies based on sex. Two more studies were similarly complicated by age as a covariant (Rosengren et al., 2011; Patterson et al., 2021). Another study was restricted to participants aged between 20 and 29 years, and found no sex difference in p1–n1 latency (Shahnaz and David, 2021). This study barely met inclusion criteria. It contained a nested study of head turn versus head raise procedures which may have affected VEMP p1–n1 amplitude measurements (Gattie et al., 2021). Latency measurements were not reported in detail, with the absence of a sex difference implied and a comment that latencies were longer than in several other studies which used shorter duration stimuli. The aim of the study was to evaluate electrode montages rather than sex difference, and it does not seem directly comparable to the current study.

Figure 15 might be proposed as supporting an interpretation in which with 300 or more presentations per participant, recruitment of more than about 44 participants is unnecessary. However, such an interpretation is not at all well supported by data. The most that could be inferred is that with exactly 48 participants, there seems little to be gained for measurements of VEMP p1–n1 latency by increasing presentation count from about 1,000 to about 3,000. At least two difficulties follow from such an inference. Firstly, the inference only applies when VEMP p1–n1 latency is the sole outcome measure. As described in sections 1 and 2, VEMP p1–n1 amplitude was also an outcome measure of interest. Assessment of VEMP p1–n1 amplitude requires a different statistical analysis (amplitude growth measurements, as reported in section 3.3) using a far greater amount of data than for VEMP latency assessment. From this perspective, the large presentation count available in the current study for analysis of VEMP latency is not an indication of excess data collection, but is rather a side effect of the need to collect a large quantity of data to accurately assess VEMP p1–n1 amplitude growth. Secondly, the current study presents no data upon which to model the effects of a participant count higher than 48. Thus, it may be that with a participant count higher than 48, studies can be adequately powered to detect a sex difference in VEMP p1–n1 latency even with fewer than 300 stimulus presentations per participant.

As regards this last-mentioned point, two further studies described in Table 1 are of interest due to their high participant counts. Brantberg et al. (2007) collected 192 VEMP responses from 1,000 participants aged between 7 and 91 years. As described by Gattie et al. (2021), the very high participant count of Brantberg et al. (2007) will have reduced an inaccuracy in electrode positioning which acts as a confounder to VEMP latency measurements. Brantberg et al. (2007) found no difference in n1 latency between women and men, however there was a significant age-sex interaction for women (*p* < 0.05) in which the p1 latency was not as prolonged with higher age for women as it was for men. The indication is therefore of VEMP p1–n1 inter peak latency becoming more prolonged for women than for men with age. Li et al. (2015) tested 186 participants aged between 26 and 92 years, but with a positively skewed age distribution (mean age was 73 years). Their age sampling was in contrast to the current study, in which all participants were aged between 16.6 and 21.1 years. Li et al. (2015) found p1 latency shortened by 0.39 ms in women compared to men (*p* = 0.005). The suggestion is of a longer p1–n1 inter peak latency for women versus men. There is again a contrast to the current study, which found a shorter p1–n1 inter peak latency for women versus men. Li et al. (2015) did not report n1 latency, making further comparison between their study and the current study difficult. However, it may be important that whereas Li et al. (2015) found p1 latency shortened by 0.39 ms in women compared to men, in the current study p1 latency was prolonged by 0.74 ms in women compared to men, albeit at the statistically insignificant *p* = 0.11 level (see section 3.2).

For both sexes, p1–n1 latency has been found to increase across the lifespan. Meta-analysis between groups older and younger than 55 years showed that in the older group, p1 was prolonged by 1.2 ms (95% CI [0.2, 2.1]) whilst n1 was prolonged by 2.8 ms (95% CI [0.3, 5.3]) (Macambira et al., 2017). The sex-specific indication from available data is that between the ages of 17–21 years (current report) p1 is prolonged and n1 shortened in women compared to men. Across the lifespan, p1 becomes prolonged in both sexes, but moreso in men than in women (Brantberg et al., 2007). When the test group has a mean age of 73 years the p1 latency is prolonged in men compared to women (Li et al., 2015), which is the opposite of the situation at ages 17–21 years (current report). Caution is warranted with this interpretation, which is based on three cross-sectional studies which sometimes have incomplete data, e.g., around age/sex matching or n1 latency. However, it is presented here as the best summary of currently available data for sex difference in VEMP p1–n1 latency.

For the reasons described by Gattie et al. (2021), evaluation of VEMP p1–n1 amplitude will require greater statistical power than evaluation of VEMP p1–n1 latency. Accordingly, only studies with more than the 44 participants and 300 stimulus presentations necessary to evaluate sex differences in p1–n1 latency should be considered when assessing sex differences in p1–n1 amplitude. Of the prior studies meeting these criteria, five found no sex difference in p1–n1 amplitude (Brantberg et al., 2007; Li et al., 2015; Rosengren et al., 2011; Carnaúba et al., 2011; Blakley and Wong, 2015). These include the only two studies with substantially more than 100 participants (Brantberg et al., 2007; Li et al., 2015). Four prior studies meeting the criteria did find a sex difference in p1–n1 amplitude. In three the p1–n1 amplitude was larger in women (Welgampola and Colebatch, 2001; Lee et al., 2008; de Oliveira Barreto et al., 2011 but only at 1,000 Hz and 2,000 Hz, not at 250 Hz, 500 Hz) and in one it was larger in men (Shahnaz and David, 2021). However all of these studies were subject to the confounders described by Gattie et al. (2021). The current report minimised the confounders described by Gattie et al. (2021), and found no sex difference in VEMP p1–n1 amplitude. Based on the current report and five prior studies (Brantberg et al., 2007; Li et al., 2015; Rosengren et al., 2011; Carnaúba et al., 2011; Blakley and Wong, 2015), the indication is of no sex difference in VEMP p1–n1 amplitude.

### 4.2 Explanations for sex difference in VEMP p1–n1 latency

The overall indication from the current study is that there is a sex difference in VEMP p1–n1 peak to trough latency that changes across the lifespan. Possible causes for the sex difference will be described here. Causes unrelated to the vestibulo-collic reflex include head and neck size, vibratory modes within the skull and the superposition of travelling and standing waves in the sternocleidomastoid muscle. Causes related to the vestibulo-collic reflex arc include peripheral and central anatomy, myelination and sex hormones. These causes are not mutually exclusive. It will also be suggested that use of BC stimuli can improve VEMP research when ear specific information is not essential, due to the greater amount of data which can be collected without exceeding safe sound exposure levels.

#### 4.2.1 Possible causation unrelated to the vestibulo-collic reflex arc

Three possible causal explanations for the finding of sex difference in VEMP p1–n1 latency are described in this section. All are based on conduction outside the vestibular system. The first involves vibratory propagation from the transducer producing stimuli to hair cells in the vestibular system. The second involves the nature of the stimuli. The third involves electromagnetic conduction from the sternocleidomastoid muscle (SCM) to electrodes placed on the surface of the skin. In all of these explanations, the shortened p1–n1 latency in women compared to men would be a side effect of difference in non-vestibular physiology (e.g., of smaller head and neck volume in women than in men), rather than a difference between women and men in function of the vestibular system itself. Explanations involving conduction outside the vestibular system are supported by studies showing a smaller size for neck circumference (Vasavada et al., 2008; Machino et al., 2021) and the temporal bone (Marcus et al., 2013). which includes the vestibular periphery. in women compared to men. Care is needed though, because explanations within and without the vestibular system are not mutually exclusive. Thus, it is possible for both types of explanation to be true. It is moreover possible for both types of explanation to be true but to have different directions of fit (e.g., one prolongs latency, the other shortens latency), and it is possible that the effect from one explanation outweighs that from the other.

At first glance, an explanation based on non-vestibular conduction appears plausible. If head and neck size are smaller in women than men, the distance over which conduction occurs is also smaller. On the basis that propagation speeds for sound/vibration and electromagnetic radiation are constant, a shorter conduction time could be expected in women than in men. The consequence would be that both p1 and n1 latencies should be shorter for women than men. However, it is not obvious that latency difference between the p1 peak and the n1 trough should be shorter in women than in men, as was found in the current study. This is because if p1 and n1 latencies are equally affected by shorter conduction times, calculation of the p1–n1 peak to trough latency by subtraction should cancel out the effect of conduction time. Consider in this regard the study of Brantberg et al. (2007), described in the appendix, which found that p1 latency became less prolonged in women than in men with increasing age, but that there was no age or sex effect on n1 latency. This describes two sets of asymmetries accompanying VEMP latency changes with increasing age: one is in p1 latency versus n1 latency, and another is in women compared to men. These asymmetries are not easily explicable using non-vestibular physiology. The reason for this is firstly that conduction effects would be expected to affect p1 and n1 equally, and secondly that men and women would be expected to be affected equally by the physiological changes which are associated with ageing (e.g., sarcopenia) (Machino et al., 2021). There is moreover an effect size difficulty with an explanation involving vibratory propagation from bone conductor to vestibular mechanoreceptors. The mastoid bone placement for the bone conductor in the current study was within approximately 10 cm of vestibular mechanoreceptors. A propagation rate of 300 m/s for vibration through the head is likely in humans (Hotehama and Nakagawa, 2012). Thus, the direct propagation time for vibration from bone conductor to vestibular hair cells is less than 0.4 ms for either men or women. However, the sex difference in p1–n1 peak to trough latency found in the current study was 2.4 ms. The propagation time from bone conductor to vestibular hair cells is in and of itself too short to explain the sex difference established in p1–n1 inter-peak latency. Based on direct propagation of vibrational energy, and not on any other consideration, there is no plausible explanation for sex difference in VEMP p1–n1 latency.

A second prospective explanation for findings based on conduction outside the vestibular system concerns the nature of stimuli. The vestibulo-collic reflex arc begins with neural impulses along the VIII cranial nerve which are created when vestibular hair cells are deflected by vibrational energy. Stimulus type is thought to have an effect on the loci of hair cells which are deflected. For example, tests in guinea pigs have shown that air conducted stimuli preferentially activate the saccule (Curthoys et al., 2006) whereas body conducted stimulation is more evenly distributed between saccule and utricle (Curthoys et al., 2016). In humans, body conducted vibration delivered to the mastoid appears to preferentially activate the utricle (Govender et al., 2015). Both saccule and utricle are otolith organs. Vestibular hair cells in a neuroepithelial layer are attached to crystalline structures in an otoconial layer, in such a way that the relative motions of the two layers will result in coordinated deflections of large numbers of vestibular hair cells, and hence coordinated variation to resting state action potential frequencies. For example, one account of the vestibular system’s response to vibration distinguishes between oscillatory modes in otoliths referred to as “accelerometer” and “seismometer” (Curthoys et al., 2019). When collecting VEMPs, two types of vibratory effect may alter the nature of hair cell deflection in the vestibular system. The first would depend on stimulus delivery (e.g., air or bone conduction, including delivery point for bone conduction). The second would involve oscillatory mode of otoliths. These effects would interact. They would moreover vary as a function of stimulus type (e.g., click or tone burst) and the vibratory modes within individual heads.

Vibratory modes within the skull are complex (Williams and Howell, 1990; Sohmer, 2017; Dobrev et al., 2017). Among several factors, vibratory modes depend upon head size. Thus, smaller head sizes in women than men could result in the oscillatory modes of otoliths differing between women and men for stimuli which are identical at the point of delivery. Suppose this biomechanical effect does occur, and that the differences in oscillatory modes alter firing rates of vestibular hair cells sufficiently to affect VEMP latency. Such a circumstance could account for the VEMP p1–n1 latency difference found between women and men in the current study. Cervical VEMPs have been recorded from click stimuli, and from tone bursts with duration of up to six milliseconds, delivered at a variety of locations (Rosengren et al., 2019). The nature of stimulation may crucially affect the response of the vestibular system. Support for this idea comes from the observation that delivery position and phase of bone conducted tone bursts affects VEMP amplitude and latency (Govender et al., 2016). Vestibular tuning has been found to depend on age (Janky and Shepard, 2009; Piker et al., 2013). It may also depend on sex. As described in the appendix, participant and presentation counts are crucial for accurate appraisal of VEMP response. It may also be that stimulus type is a crucial factor when women and men are compared.

The third and final causal explanation based on conduction outside the vestibular system concerns the change in electromagnetic field created by the cessation in muscle fibre activity in the sternocleidomastoid muscle (SCM) during the vestibulo-collic reflex. Recordings from electrodes on the surface of the skin capture a compound muscle activation potential from many muscle fibres (Kane and Oware, 2012; Mallik and Weir, 2005). This summed activity is subject to volume conduction (Rutkove, 2007; Farina et al., 2002), a phenomenon in which the combined electromagnetic activity from many individual muscle fibres, along with electromagnetic propagation through non-excitable tissue (notably subcutaneous fat; Bartuzi et al., 2010) shapes electromagnetic potentials recorded on the skin surface. Thicknesses of the SCM and subcutaneous tissue have been measured with sonography (Chang et al., 2007), with the finding that raw amplitudes of VEMPs in adults correlated negatively with subcutaneous thickness. Despite this, the relationship with thickness of both subcutaneous tissue and SCM became statistically insignificant when VEMP amplitudes were corrected for pre-stimulus EMG background, as was the case in the current study. Thus, volume conduction appears at first glance unlikely to have been an appreciable factor affecting VEMP measurements.

However, there are two complications concerning electromagnetic conduction through the SCM. Both are related to neck length. Firstly, if electrodes were not positioned at the shortest possible distance from the electromagnetic source within the SCM, volume conduction will have increased. Electrode placement for the current study was via a clinical palpation technique (British Society of Audiology, 2012) which was consistent between women and men. The palpation technique aimed to position the electrode directly above the belly of the SCM. Locating the belly of the SCM via palpation will have become less accurate as a function of SCM size, which will have varied in turn as a function of neck length. Thus, a neck length difference between women and men could have led to a variation in electrode positioning which resulted in a sex difference in VEMP measurements, as was found in the current study.

The second complication concerns the nature of the VEMP. The VEMP has been described as a superposition of motor unit action potentials (Wit and Kingma, 2006; Lütkenhöner, 2019). Consider in this regard the proposition that the VEMP is a combination of several wave forms, including a standing wave and a travelling wave, which originate at a central motor point and propagate along the SCM in both directions (Rosengren et al., 2016). In such a scenario, a difference in SCM length between women and men could lead to a sex difference in standing waves and, through superposition with travelling waves, an alteration to VEMP p1–n1 latency as was found in the current study. Similar considerations will follow for other models of the VEMP involving superposition (Wit and Kingma, 2006; Lütkenhöner, 2019; Wei et al., 2013). Neck length once again emerges as an important factor.

In anthropometrical comparison, neck length in women has been found to be no different from men between the sternum and tragus, but 7 mm shorter between the C7 spinous process and tragus (Vasavada et al., 2008). However, these data were collected to assess sex difference pertaining to whiplash injury. As such, participants were closely paired on height and neck length, with an initial sample of 90 screened down to 28. Even then, neck variation could be substantial (e.g., 20 mm longer for the man than the woman in one pair). Another set of neck length data, from 88 participants, showed that the distance between the C7 spinous process and the external occipital protuberance was 9.5 mm shorter in women than men (Ahmed et al., 2020). The sex comparison was only made for participants with spondylosis, although comparison with non-spondlyosis controls showed that neck length was not a statistically significant predictor for spondylosis.

VEMPs have been compared between children and adults. The averaged findings were that in adults necks were 38 mm longer (mastoid tip to clavicle), p1 latency was 2.9 ms longer, n1 latency was 3.1 ms longer, and p1–n1 latency was 0.9 ms longer (Chang et al., 2007). Male and female necks were not measured in the current study. If male necks were longer than female necks then VEMP latencies were prolonged with increased neck length, similar to adults versus children. However, effect sizes differed: 1.7 ms for n1, and 2.4 ms for p1–n1 for men versus women in the current study, compared to 3.1 ms for n1 and 0.9 ms for p1–n1 for adults compared to children. Other relevant studies evaluated the effect of deliberately positioning active electrodes away from the belly of the SCM (Rosengren et al., 2016; Ashford et al., 2016). Rosengren et al. (2016) found that an active electrode position 50 mm from the belly of the SCM prolonged p1 and n1 absolute VEMP latencies by 3 ms. Needle electrode data were reported in integer milliseconds only, but surface electrode data for the same location were reported in sub-milliseconds, and show a prolongation of 2.3 ms in VEMP p1–n1 latency. These data are comparable to the 1.7 ms prolongation in VEMP n1 latency, and 2.4 ms prolongation in VEMP p1–n1 latency, found in the current study for men compared to women. Rosengren et al. (2016), tested three women and three men, which provided insufficient data for statistical comparison based on sex. However, the range of measurement in their study appears quite wide (e.g., the middle surface electrode n1 latency of 21.8 ms had a range of 17.4–28.0 ms, whilst a single motor unit needle electrode at the same location showed a latency of 12 ms with a range of 9–21 ms). This raises the possibility that changing electrode positioning had differing effects on VEMP latency between women and men, and that such effects could have become apparent with a larger participant count.

Overall, there are too few data to draw a conclusion on whether differences between female and male necks could have contributed to the VEMP latency differences found in the current study. The indication is that volume conduction is unlikely to have been an appreciable factor. However, until more is understood of the precise nature by which the VEMP originates within and spreads throughout the SCM, the possibility of apparently small changes in neck dimensions having an appreciable effect on VEMP latency measurements cannot be ruled out.

#### 4.2.2 Possible causation affecting the vestibulo-collic reflex arc

Another possible cause for the finding of a sex difference in VEMP p1–n1 latency may be a structural or functional difference between women and men which manifests in the vestibular reflex arc described in Figure 1. Such explanations will consider activity in the vestibular periphery, the VIII and XI cranial nerves, the vestibular nucleus, the medial vestibulospinal tract and the sternocleidomastoid (SCM). Several of these areas were featured in the review of Smith et al. (2019). Relevant findings are summarised and extended here.

Quantities of type I and type II vestibular hair cells have not been found to differ significantly between men and women in any of the vestibular sensory organs (Merchant et al., 2000). Vestibular hair cells innervate, and for type II cells are innervated by, the bipolar neurons in Scarpa’s ganglion. Women have been found to have fewer Scarpa’s ganglion neurons than men (Velázquez-Villaseñor et al., 2000). Axons from Scarpa’s ganglion neurons comprise the vestibular portion of the VIII cranial nerve. Morphometric analysis of Scarpa’s ganglion axons has found no difference between women and men in average transverse area, and significantly fewer myelinated axons in women than in men (18,022 compared with 21,006; Moriyama et al., 2007). Other than the X cranial nerve, the vestibular portion of the VIII cranial nerve was the only one of 13 peripheral nerves to show a sex difference in a morphometric comparison (Moriyama et al., 2016). However, the XI cranial nerve, which innervates the sternocleidomastoid, was not included in the morphometric comparison, and on available data can only be evaluated for sex difference using a conduction study (Cleavenger et al., 2019). Latencies were assessed between the C7 spinous process and the trapezius muscle, in which the XI cranial nerve terminates after branching to the sternocleidomastoid. Latencies were up to 0.4 ms shorter in women than in men. This is too small a difference to account for the finding of the current study that VEMP p1–n1 latency was shorter in women than men by 2.4 ms.

Sex differences have been established in central components of the vestibular system. The vestibular system projects directly to cerebellar vermis (Hitier et al., 2014). Several studies have found that cerebellar vermis is larger in men than women (Raz et al., 1992, 1998, 2001; Tiemeier et al., 2010), although sometimes the opposite has been found (Luft et al., 1999) and some studies have found no group difference (Rhyu et al., 1999; Bernard et al., 2015; Metwally et al., 2021). Vestibular nuclei span the pons and medulla, which have been investigated in humans using diffusion tensor imaging (Bouhrara et al., 2021). Sex differences were found in the pons but not the medulla, via measures of fractional anisotropy, mean diffusivity and radial diffusivity. Measures of axial diffusivity, longitudinal and transverse relaxation rates, and myelin water fraction showed no sex difference. Ayyildiz et al. (2008) found the right medial vestibular nucleus (MVN) had a larger volume when comparing female to male rats. They also found a laterality difference, with male rats having more neurons in the left than the right MVN, and female rats having more neurons in the right than the left MVN.

In the brainstem generally, as in the cerebrum, the indication is that women have higher myelin content than men (Arshad et al., 2016; Bouhrara et al., 2020; Bouhrara et al., 2021). This is consistent with an interpretation in which proliferation of neuroglia and myelin proteins are regulated differently in women compared to men (Cerghet et al., 2006; Greer et al., 2004). Such a difference in regulation may be due to sex hormones (Marin-Husstege et al., 2004; Cerghet et al., 2009; Schumacher et al., 2012). Sex hormones were proposed by Ayyildiz et al. (2008) to play a crucial role in the sexual dimorphism they found in the MVN, with the contribution being primarily neurodevelopmental. Smith et al. (2019) reviewed a variety of studies demonstrating that the sex hormone oestrogen has an effect on brain areas considered to be important for the vestibular system, along with studies showing that chemicals known to be toxic to the vestibular system have a differing effect in female versus male animals. They also highlighted the shortage of studies appraising testosterone levels. A similar observation has been made by researchers in endocrinology (Singh Ospina et al., 2015), since androgens as well as oestrogens have been found to regulate critical biological and pathological processes in both males and females (Hammes and Levin, 2019). For example, injection of testosterone propionate has been found to reduce the effect of immune-mediated sensorineural hearing loss in female Wistar rats (Yeo et al., 2003). In male Long Evans Hooded rats, vestibular dysfunction caused by repeated mild traumatic brain injury was reduced in rats receiving testosterone, with the treatment improving vestibular neuronal survival by comparison to a sham rat group (Foecking et al., 2022). Testosterone has been found to mediate synaptic responses in MVN slices from male rats, depending on its conversion to oestrogenic or androgenic metabolites (Grassi et al., 2010; Scarduzio et al., 2013), with vestibular synaptic transmission depending on oestrous cycle in MVN slices from female rats (Pettorossi et al., 2011; Grassi et al., 2012). In humans, sex hormones have been linked to vestibular migraine, vertigo and Meniere’s disease (Tang et al., 2021; Park and Viirre, 2010; Seemungal et al., 2001).

### 4.3 BC stimuli as a way to increase statistical power

AC stimuli for VEMP testing are preferable to BC stimuli when ear specific information is required. This is due to intracranial conduction in which BC stimuli necessarily evoke a response from both ears, invalidating ear specific measures. This limitation must be considered against the risk to hearing health when using high amplitude AC stimulation. The greater amount of data which can be safely collected with BC stimuli may be preferred to the ear specific measures available with AC stimuli for some VEMP study designs.

Concerns around safe AC stimulus levels may even be understated, due to a potential underestimation of sound pressure level (SPL) at the tympanic membrane when variation in ear canal size is considered. Standardised calibration procedures (e.g., following ANSI S3.7) use a 2 cc coupler. Correction for ear canals with volumes other than 2 cc can be carried out on the basis that pressure is inversely proportional to volume (Boyle, 1662). From the definition of the dB scale, and using a 2 cc cavity as the reference, the necessary dB adjustment to dial SPL is 20 times the base 10 logarithm of the ratio of the 2 cc coupler volume to the actual ear canal volume. Conveniently, it is not necessary to get involved in any actual pressure calculations. Rather, corrections can be made by addition or subtraction using log arithmetic, transforming the dB denominated pressure value relative to the 20 μPa standard. Thus, for an ear canal size of 1 cc, the correction is 20 log(2), or 6.02 dB. The correction for other ear canal volumes follows a similar arithmetic. For example, an ear canal size of 0.7 cc (not atypical for a woman) requires an increase to the dial SPL of 20 log (2/0.7), or 9.1 dB. Ear canal size for both sexes has a 90% range between approximately 0.6 and 1.5 cc (Margolis and Heller, 1987). Thus, dial settings for dB (HL or SPL) may be an appreciable underestimate when equipment is calibrated with a 2 cc coupler. This consideration is borne out in the study of Thomas et al. (2017), who measured a 0.29 cc difference between ear canal sizes of differently aged child groups using tympanometry. They found this corresponded to an approximate 3 dB difference in ear canal sound pressure as recorded with a probe microphone, which compares to a predicted 2.9 dB difference using the formula just described.

Based on an average ear canal size of 1 cc, 600 presentations of an AC 0–1-0 500 Hz tone burst (i.e., rise/fall time of zero and plateau of 2 ms) over insert earphones would exceed EU safe sound exposure levels at the typical 100 dB nHL levels used to elicit VEMP responses (Portnuff et al., 2017). Whereas with BC stimuli as used in the current report, a total of 50,000 presentations of a 0–1-0 500 Hz tone burst would amount to less than 80% of EU safe sound exposure levels. There is effectively no restriction based on safe sound levels at the cochlea for the amount of VEMP data which could be collected in single session using BC stimuli.

### 4.4 Clinical implications

Concerns over safe sound levels for VEMP testing (i.e., as per Mattingly et al., 2015; Portnuff et al., 2017; Asakura and Kamogashira, 2021) can be assuaged by using BC rather than AC stimuli. An additional benefit of BC stimuli is that they enable a more precise appraisal of VEMP than AC stimuli. Thus, BC stimuli may be preferred for initial appraisal in the clinic, with AC testing following only when precise diagnostic detail is required. This might, for example, be ear-specific information or deliberate use of AC for focal targeting of utricle/saccule.

VEMP latency has clinical relevance to assessment of conditions such as benign paroxysmal positional vertigo (Oya et al., 2019), multiple scleroris (Gür et al., 2022), recurrent vertigo of childhood (Gao et al., 2022) and fibromyalgia (Bayazit et al., 2010). The finding of a sex difference in VEMP p1–n1 latency could be important in establishing normative values for diagnostic tests. It has particular relevance due to the higher rate of diagnosis of vestibular-related conditions in women than men (Smith et al., 2019). These include benign paroxysmal positional vertigo, vestibular vertigo, vestibular neuritis, vestibular migraine, mal de debarquement syndrome, unspecified peripheral vestibular dizziness and motion sickness (Hülse et al., 2019; Hain and Cherchi, 2016; Cha et al., 2018). As discussed in section 4.2.2, influence of sex hormones on myelination or synaptic response may underlie findings of sex difference in VEMP latency.

### 4.5 Comparison to animal studies

Raciti et al. (2023) conducted a study with Brown Norway rats that had several similarities to the current study. One similarity was that neck tension was precisely controlled, either using a custom rodent holder (Raciti et al., 2023) or a headbar with biofeedback (current study). Another similarity was the large quantity of data collected, either by conducting a thorough characterization of VEMP in an animal model using multiple frequencies (Raciti et al., 2023) or by presenting a large quantity of stimuli to humans safely using BC (current study). Yet another similarity was in the age and health of the animals studied, which were rats aged 14–18 weeks (Raciti et al., 2023) or humans aged 16–21 years (current study) with no health issues identified in any group tested.

Raciti et al. (2023) found a sex difference in p1 latency. This was always shortened in male compared to female rats, and the difference reached statistical significance for all frequencies tested between 6–16 kHz (the total frequency range tested was 2–16 kHz in 2 kHz steps, plus a 1 kHz test). Raciti et al. (2023) did not report n1, so a direct comparison with the p1-n1 latency measure in the current study is not possible. However, the finding was similar to that reported in section 3.2, in which p1 latency was shortened by 0.74 ms in men compared to women. Although this did not reach statistical significance (*p* = 0.11) (i.e., unlike the findings of significantly shorter n1 latency (1.69 ms, *p* = 0.015), and p1-n1 interpeak latency (2.43 ms, *p* = 0.0020) in women compared to men) the data reported by Raciti et al. (2023) were consistent with findings in the current study. This included the findings of both Raciti et al. (2023) and the current study that there was no sex difference in VEMP amplitude. Raciti et al. (2023) included reliability and replicability tests supporting their findings.

Dissimilarities between Raciti et al. (2023) and the current study are important. Use of AC stimuli by Raciti et al. (2023) may have preferentially stimulated the saccule (Curthoys and Grant, 2015), whereas the BC stimuli used in the current study are likely to have had a more even effect between saccule and utricle. This need not necessarily have created a functional difference in the reflex arc that is measured during VEMP recording (see Figure 1), however such a possibility should be evaluated in follow up studies. Another dissimilarity was between the rat and human; follow up with other species will be necessary to establish whether sex difference in VEMP latency is consistent across vertebrates.

## 5 Conclusion

The clinical VEMP protocol for humans was adapted to increase sensitivity. Exclusive use of body-conducted stimuli removed concerns around exceeding safe sound exposure levels with the high amplitude air-conducted stimuli required for VEMP testing, thereby enabling collection of a greater amount of data than would have been achievable using air-conducted stimuli. This was combined with other methods of increasing statistical power, including use of a padded headbar and biofeedback to control sternocleidomastoid tension, and linear mixed effects modelling of VEMP amplitude growth.

This protocol led to the finding that among participants with a mean age of 19.5 years, VEMP p1–n1 latency was approximately 20% shorter in women than in men. There was no difference between women and men in VEMP p1–n1 amplitude. The latency finding is consistent with a preclinical model using a similar protocol (precise control of sternocleidomastoid tension, collection of larger quantities of data than standardly achievable in human clinical VEMP testing) with Brown Norway rats (Raciti et al., 2023). It is a reversal of the overall indication of no sex difference from 15 prior studies in humans. The prior studies were reviewed, with a power analysis suggesting they were underpowered to detect the sex difference.

Several candidate explanations for sex difference in VEMP latency were identified. These included the influence of sex hormones, which may have affected myelination or synaptic response, and conduction effects based around head/neck size. These possibilities are neither exhaustive nor mutually exclusive. Rather, the factors described could operate simultaneously, with some effects outweighing others in an aggregate measure such as VEMP latency. The interactions may be age-dependent, for example due to co-variation with alteration to muscle tone or levels of sex hormones. Such an interpretation is supported by the indication from literature review that the nature of the sex difference in VEMP p1–n1 latency changes across the lifespan. The overall indication is that normative values for VEMP p1–n1 latency will be both sex and age dependent.

## Data Availability

Electrophysiological data and R scripts are available for download from the authors upon request.

## References

[ref2] Ahmed S. B. Qamar A. Imram M. Fahim M. F. (2020). Comparison of neck length, relative neck length and height with incidence of cervical spondylosis. Pak. J. Med. Sci. 36, 219–223. doi: 10.12669/pjms.36.2.832, PMID: 32063963 PMC6994911

[ref3] Akin F. W. Murnane O. D. Proffitt T. M. (2003). The effects of click and tone-burst stimulus parameters on the vestibular evoked myogenic potential (VEMP). J. Am. Acad. Audiol. 14, 500–509. doi: 10.3766/jaaa.14.9.5 14708838

[ref4] Arshad M. Stanley J. A. Raz N. (2016). Adult age differences in subcortical myelin content are consistent with protracted myelination and unrelated to diffusion tensor imaging indices. NeuroImage 143, 26–39. doi: 10.1016/j.neuroimage.2016.08.047, PMID: 27561713 PMC5124541

[ref5] Asakura S. Kamogashira T. (2021). Sudden bilateral hearing loss after vestibular-evoked myogenic potentials. Clin. Case Rep. 9:e05025. doi: 10.1002/ccr3.5025, PMID: 34849224 PMC8607976

[ref6] Ashford A. Huang J. Zhang C. Wei W. Mustain W. Eby T.. (2016). The cervical vestibular-evoked myogenic potentials (cVEMPs) recorded along the sternocleidomastoid muscles during Head rotation and flexion in Normal human subjects. J. Assoc. Res. Otolaryngol. 17, 303–311. doi: 10.1007/s10162-016-0566-8, PMID: 27105980 PMC4940286

[ref7] Ayyildiz M. Kozan R. Agar E. Kaplan S. (2008). Sexual dimorphism in the medial vestibular nucleus of adult rats: stereological study. Anat. Sci. Int. 83, 131–139. doi: 10.1111/j.1447-073X.2007.00220.x, PMID: 18956784

[ref8] Balduzzi S. Rücker G. Schwarzer G. (2019). How to perform a meta-analysis with R: a practical tutorial. Evid. Based Ment. Health 22, 153–160. doi: 10.1136/ebmental-2019-300117, PMID: 31563865 PMC10231495

[ref9] Barr D. J. Levy R. Scheepers C. Tily H. J. (2013). Random effects structure for confirmatory hypothesis testing: keep it maximal. J. Mem. Lang. 68, 255–278. doi: 10.1016/j.jml.2012.11.001, PMID: 24403724 PMC3881361

[ref10] Bartuzi P. Tokarski T. Roman-Liu D. (2010). The effect of the fatty tissue on EMG signal in young women. Acta Bioeng. Biomech., 12, 87–92. 20882946

[ref11] Basta D. Todt I. Ernst A. (2005). Normative data for P1/N1-latencies of vestibular evoked myogenic potentials induced by air- or bone-conducted tone bursts. Clin. Neurophysiol. 116, 2216–2219. doi: 10.1016/j.clinph.2005.06.010, PMID: 16043396

[ref12] Basta D. Todt I. Ernst A. (2007). Characterization of age-related changes in vestibular evoked myogenic potentials. J. Vestib. Res. 17, 93–98. doi: 10.3233/VES-2007-172-304, PMID: 18413902

[ref13] Bates D. Mächler M. Bolker B. Walker S. (2015). Fitting Linear Mixed-Effects Models Using lme4. Journal of Statistical Software 67, 1–48. doi: 10.18637/jss.v067.i01, PMID: 24403724

[ref14] Bayazit Y. A. Celenk F. Gunduz A. G. Gunduz B. Ondag N. Meray J. (2010). Vestibular evoked myogenic potentials in patients with fibromyalgia syndrome. J. Laryngol. Otol. 124, 610–615. doi: 10.1017/S0022215110000010, PMID: 20170583

[ref15] Bernard J. A. Leopold D. R. Calhoun V. D. Mittal V. A. (2015). Regional cerebellar volume and cognitive function from adolescence to late middle age. Hum. Brain Mapp. 36, 1102–1120. doi: 10.1002/hbm.22690, PMID: 25395058 PMC4323630

[ref17] Blakley B. W. Wong V. (2015). Normal values for cervical vestibular-evoked myogenic potentials. Otol. Neurotol. 36, 1069–1073. doi: 10.1097/MAO.0000000000000752, PMID: 25839981

[ref18] Bouhrara M. Cortina L. E. Khattar N. Rejimon A. C. Ajamu S. Cezayirli D. S.. (2021). Maturation and degeneration of the human brainstem across the adult lifespan. Aging 13, 14862–14891. doi: 10.18632/aging.203183, PMID: 34115614 PMC8221341

[ref19] Bouhrara M. Rejimon A. C. Cortina L. E. Khattar N. Bergeron C. M. Ferrucci L.. (2020). Adult brain aging investigated using BMC-mcDESPOT-based myelin water fraction imaging. Neurobiol. Aging 85, 131–139. doi: 10.1016/j.neurobiolaging.2019.10.003, PMID: 31735379 PMC6924176

[ref20] Brantberg K. Fransson P. A. (2001). Symmetry measures of vestibular evoked myogenic potentials using objective detection criteria. Scand. Audiol. 30, 189–196. doi: 10.1080/010503901316914566, PMID: 11683457

[ref21] Brantberg K. Granath K. Schart N. (2007). Age-related changes in vestibular evoked myogenic potentials. Audiol. Neurootol. 12, 247–253. doi: 10.1159/000101332, PMID: 17389791

[ref22] British Society of Audiology (2012). Performing cervical vestibular evoked myogenic potential measurements. Available online at: https://www.thebsa.org.uk/wp-content/uploads/2014/04/VEMP_Guidance_v1.1_20121.pdf (Accessed July 2014).

[ref23] British Society of Audiology (2014). Recommended procedure: Tympanometry. Available online at: www.thebsa.org.uk (Accessed September 2022).

[ref24] British Society of Audiology (2018). Recommended procedure: Pure-tone air-conduction and bone- conduction threshold audiometry with and without masking. Available online at: www.thebsa.org.uk (Accessed September 2022).

[ref25] British Society of Audiology (2022). Recommended procedure: Ear examination. Available online at: www.thebsa.org.uk

[ref26] Burkard R. (2006). Calibration of acoustic transients. Brain Res. 1091, 27–31. doi: 10.1016/j.brainres.2006.02.132, PMID: 16631624

[ref27] Carnaúba A. T. L. Farias V. V. Santos N. Oliveira A. C. D. Rodrigues R. G. D. S. Menezes P. D. L. (2011). Influence of gender on the vestibular evoked myogenic potential. Braz. J. Otorhinolaryngol. 77, 245–248. doi: 10.1590/S1808-86942011000200015 21537627 PMC9452196

[ref28] Cerghet M. Skoff R. P. Bessert D. Zhang Z. Mullins C. Ghandour M. S. (2006). Proliferation and death of oligodendrocytes and myelin proteins are differentially regulated in male and female rodents. J. Neurosci. 26, 1439–1447. doi: 10.1523/JNEUROSCI.2219-05.2006, PMID: 16452667 PMC6675481

[ref29] Cerghet M. Skoff R. P. Swamydas M. Bessert D. (2009). Sexual dimorphism in the white matter of rodents. J. Neurol. Sci. 286, 76–80. doi: 10.1016/j.jns.2009.06.039, PMID: 19625027 PMC2760672

[ref30] Cha Y. H. Cui Y. Y. Baloh R. W. (2018). Comprehensive clinical profile of mal De Debarquement syndrome. Front. Neurol. 9:261. doi: 10.3389/fneur.2018.00261, PMID: 29867709 PMC5950831

[ref31] Chang C.-H. Yang T.-L. Wang C.-T. Young Y.-H. (2007). Measuring neck structures in relation to vestibular evoked myogenic potentials. Clin. Neurophysiol. 118, 1105–1109. doi: 10.1016/j.clinph.2007.01.020, PMID: 17368089

[ref32] Cleavenger A. Dean H. Foster R. George K. Hotle B. Lewis K.. (2019). Gender, BMI and side-to-side differences in spinal accessory nerve conduction from the upper and middle components of the trapezius muscle. J. Bodyw. Mov. Ther. 23, 588–593. doi: 10.1016/j.jbmt.2019.04.009, PMID: 31563375

[ref33] Cohen J. (1969). Statistical power analysis for the behavioral sciences. Available online at: https://play.google.com/store/books/details?id=rEe0BQAAQBAJ&source=gbs_api

[ref35] Colebatch J. G. Halmagyi G. M. (1992). Vestibular evoked potentials in human neck muscles before and after unilateral vestibular deafferentation. Neurology 42, 1635–1636. doi: 10.1212/WNL.42.8.1635, PMID: 1641165

[ref36] Colebatch J. G. Rosengren S. M. Welgampola M. S. (2016). Vestibular-evoked myogenic potentials. Handb. Clin. Neurol. 137, 133–155. doi: 10.1016/B978-0-444-63437-5.00010-8, PMID: 27638068

[ref38] Corneil B. D. Camp A. J. (2018). Animal models of vestibular evoked myogenic potentials: the past, present, and future. Front. Neurol. 9:489. doi: 10.3389/fneur.2018.00489, PMID: 29988517 PMC6026641

[ref39] Curthoys I. S. Grant J. W. (2015). How does high-frequency sound or vibration activate vestibular receptors? Exp. Brain Res. 233, 691–699. doi: 10.1007/s00221-014-4192-6, PMID: 25567092

[ref40] Curthoys I. S. Grant J. W. Pastras C. J. Brown D. J. Burgess A. M. Brichta A. M.. (2019). A review of mechanical and synaptic processes in otolith transduction of sound and vibration for clinical VEMP testing. J. Neurophysiol. 122, 259–276. doi: 10.1152/jn.00031.2019, PMID: 31042414

[ref41] Curthoys I. S. Kim J. McPhedran S. K. Camp A. J. (2006). Bone conducted vibration selectively activates irregular primary otolithic vestibular neurons in the guinea pig. Exp. Brain Res. 175, 256–267. doi: 10.1007/s00221-006-0544-1, PMID: 16761136

[ref42] Curthoys I. S. Vulovic V. Burgess A. M. Sokolic L. Goonetilleke S. C. (2016). The response of guinea pig primary utricular and saccular irregular neurons to bone-conducted vibration (BCV) and air-conducted sound (ACS). Hear. Res. 331, 131–143. doi: 10.1016/j.heares.2015.10.019, PMID: 26626360

[ref43] de Oliveira Barreto A. C. Colafêmina J. F. de Lemos Menezes P. (2011). Saccular sensitivity function measured by vestibular evoked myogenic potential. Acta Otolaryngol. 131, 618–623. doi: 10.3109/00016489.2010.545186, PMID: 21319943

[ref44] Dobrev I. Sim J. H. Stenfelt S. Ihrle S. Gerig R. Pfiffner F.. (2017). Sound wave propagation on the human skull surface with bone conduction stimulation. Hear. Res. 355, 1–13. doi: 10.1016/j.heares.2017.07.005, PMID: 28964568

[ref45] Farina D. Cescon C. Merletti R. (2002). Influence of anatomical, physical, and detection-system parameters on surface EMG. Biol. Cybern. 86, 445–456. doi: 10.1007/s00422-002-0309-2, PMID: 12111273

[ref46] Faul F. Erdfelder E. Lang A. G. Buchner A. (2007). G*power 3: a flexible statistical power analysis program for the social, behavioral, and biomedical sciences. Behav. Res. Methods 39, 175–191. doi: 10.3758/bf03193146, PMID: 17695343

[ref47] Foecking E. M. Segismundo A. B. Lotesto K. M. Westfall E. J. Bolduan A. J. Peter T. K.. (2022). Testosterone treatment restores vestibular function by enhancing neuronal survival in an experimental closed-head repetitive mild traumatic brain injury model. Behav. Brain Res. 433:113998. doi: 10.1016/j.bbr.2022.113998, PMID: 35809692

[ref48] Forbes P. A. Dakin C. J. Vardy A. N. Happee R. Siegmund G. P. Schouten A. C.. (2013). Frequency response of vestibular reflexes in neck, back, and lower limb muscles. J. Neurophysiol. 110, 1869–1881. doi: 10.1152/jn.00196.2013, PMID: 23904494

[ref49] Forbes P. A. Fice J. B. Siegmund G. P. Blouin J.-S. (2018). Electrical vestibular stimuli evoke robust muscle activity in deep and superficial neck muscles in humans. Front. Neurol. 9:535. doi: 10.3389/fneur.2018.00535, PMID: 30026725 PMC6041388

[ref50] Fritzsch B. Pan N. Jahan I. Duncan J. S. Kopecky B. J. Elliott K. L.. (2013). Evolution and development of the tetrapod auditory system: an organ of Corti-centric perspective. Evol. Dev. 15, 63–79. doi: 10.1111/ede.12015, PMID: 23331918 PMC3918746

[ref51] Fritzsch B. Straka H. (2014). (2014). Evolution of vertebrate mechanosensory hair cells and inner ears: toward identifying stimuli that select mutation driven altered morphologies. J. Comp. Physiol. A Neuroethol. Sens. Neural Behav. Physiol. 200, 5–18. doi: 10.1007/s00359-013-0865-z, PMID: 24281353 PMC3918741

[ref52] Gao D. Sun X. Shen J. Ma X. Wang L. Chen X.. (2022). Clinical characteristics of vestibular evoked myogenic potentials in children with recurrent vertigo of childhood. Int. J. Pediatr. Otorhinolaryngol. 161:111257:111257. doi: 10.1016/j.ijporl.2022.111257, PMID: 35988372

[ref53] Gattie M. Howell P. Kluk K. Lieven E. (2019). Vestibular evoked myogenic potentials in persistent developmental stuttering. Retrieved from osf.io/9mukg 111257

[ref54] Gattie M. Lieven E. V. M. Kluk K. (2021). Weak Vestibular Response in Persistent Developmental Stuttering. Front. Integr. Neurosci. 15:662127. doi: 10.3389/fnint.2021.662127, PMID: 34594189 PMC8477904

[ref55] Gattie M. Lieven E. Kluk K. (2024). Adult stuttering prevalence II: recalculation, subgrouping and estimate of stuttering community engagement. J. Fluen. Disord. 83:106086. doi: 10.1016/j.jfludis.2024.106086, PMID: 39706110

[ref56] Gazioglu S. Boz C. (2012). Ocular and cervical vestibular evoked myogenic potentials in multiple sclerosis patients. Clin. Neurophysiol. 123, 1872–1879. doi: 10.1016/j.clinph.2012.01.022, PMID: 22418590

[ref57] Govender S. Dennis D. L. Colebatch J. G. (2015). Vestibular evoked myogenic potentials (VEMPs) evoked by air- and bone-conducted stimuli in vestibular neuritis. Clin. Neurophysiol. 126, 2004–2013. doi: 10.1016/j.clinph.2014.12.029, PMID: 25704871

[ref58] Govender S. Rosengren S. M. Dennis D. L. Lim L. J. Colebatch J. G. (2016). Contrasting phase effects on vestibular evoked myogenic potentials (VEMPs) produced by air- and bone-conducted stimuli. Exp. Brain Res. 234, 141–149. doi: 10.1007/s00221-015-4441-3, PMID: 26403294

[ref59] Grassi S. Frondaroli A. Di Mauro M. Pettorossi V. E. (2010). Influence of testosterone on synaptic transmission in the rat medial vestibular nuclei: estrogenic and androgenic effects. Neuroscience 171, 666–676. doi: 10.1016/j.neuroscience.2010.09.035, PMID: 20884332

[ref60] Grassi S. Frondaroli A. Scarduzio M. Dieni C. V. Brecchia G. Boiti C.. (2012). Influence of sex and estrous cycle on synaptic responses of the medial vestibular nuclei in rats: role of circulating 17β-estradiol. Brain Res. Bull. 87, 319–327. doi: 10.1016/j.brainresbull.2011.11.008, PMID: 22127323

[ref61] Green P. MacLeod C. J. (2016). SIMR: an R package for power analysis of generalized linear mixed models by simulation. Methods Ecol. Evol. 7, 493–498. doi: 10.1111/2041-210x.12504

[ref62] Greer J. M. Csurhes P. A. Pender M. P. McCombe P. A. (2004). Effect of gender on T-cell proliferative responses to myelin proteolipid protein antigens in patients with multiple sclerosis and controls. J. Autoimmun. 22, 345–352. doi: 10.1016/j.jaut.2004.03.004, PMID: 15120759

[ref63] Gür E. Binkhamis G. Kluk K. (2022). Effects of multiple sclerosis on the audio-vestibular system: a systematic review. BMJ Open 12:e060540. doi: 10.1136/bmjopen-2021-060540, PMID: 35977771 PMC9389089

[ref64] Hain T. C. Cherchi M. (2016). Mal de débarquement syndrome. Handb. Clin. Neurol. 137, 391–395. doi: 10.1016/B978-0-444-63437-5.00028-5, PMID: 27638086

[ref65] Hammes S. R. Levin E. R. (2019). Impact of estrogens in males and androgens in females. J. Clin. Invest. 129, 1818–1826. doi: 10.1172/JCI125755, PMID: 31042159 PMC6486327

[ref66] Heidari S. Babor T. F. De Castro P. Tort S. Curno M. (2016). Sex and gender equity in research: rationale for the SAGER guidelines and recommended use. Res. Peer Rev. 1, 2–9. doi: 10.1186/s41073-016-0007-6, PMID: 29451543 PMC5793986

[ref67] Hitier M. Besnard S. Smith P. F. (2014). Vestibular pathways involved in cognition. Front. Integr. Neurosci. 8:59. doi: 10.3389/fnint.2014.00059, PMID: 25100954 PMC4107830

[ref68] Hoenig J. M. Heisey D. M. (2001). The abuse of power: the pervasive fallacy of power calculations for data analysis. Am. Stat. 55, 19–24. doi: 10.1198/000313001300339897

[ref69] Hotehama T. Nakagawa S. (2012). Propagation velocity of bone-conducted ultrasound in the human head. Jpn. J. Appl. Phys. 51:07GF21. doi: 10.1143/JJAP.51.07GF21

[ref70] Hülse R. Biesdorf A. Hörmann K. Stuck B. Erhart M. Hülse M.. (2019). Peripheral vestibular disorders: an epidemiologic survey in 70 million individuals. Otol. Neurotol. 40, 88–95. doi: 10.1097/MAO.0000000000002013, PMID: 30289843

[ref71] Janky K. L. Shepard N. (2009). Vestibular evoked myogenic potential (VEMP) testing: normative threshold response curves and effects of age. J. Am. Acad. Audiol., 20, 514–522. Available online at: http://eutils.ncbi.nlm.nih.gov/entrez/eutils/elink.fcgi?dbfrom=pubmed&id=19764171&retmode=ref&cmd=prlinks 19764171 10.3766/jaaa.20.8.6 PMC2749261

[ref72] Kane N. M. Oware A. (2012). Nerve conduction and electromyography studies. J. Neurol. 259, 1502–1508. doi: 10.1007/s00415-012-6497-3 22614870

[ref73] Khan F. K. Balraj A. Lepcha A. (2014). Normative data for vestibular evoked myogenic potential in different age groups among a heterogeneous Indian population. Indian J. Otolaryngol. Head Neck Surg. 66, 149–154. doi: 10.1007/s12070-013-0685-z, PMID: 24822153 PMC4016341

[ref74] Kim S. Lee H. S. Kim J. S. (2010). Medial vestibulospinal tract lesions impair sacculo-collic reflexes. J. Neurol. 257, 825–832. doi: 10.1007/s00415-009-5427-5, PMID: 20054695

[ref75] Kopecky B. Santi P. Johnson S. Schmitz H. Fritzsch B. (2011). Conditional deletion of N-Myc disrupts neurosensory and non-sensory development of the ear. Dev. Dyn. 240, 1373–1390. doi: 10.1002/dvdy.22620, PMID: 21448975 PMC3092837

[ref76] Kumle L. Võ M. L. H. Draschkow D. (2021). Estimating power in (generalized) linear mixed models: an open introduction and tutorial in R. Behav. Res. Methods 53, 2528–2543. doi: 10.3758/s13428-021-01546-0, PMID: 33954914 PMC8613146

[ref77] Lakens D. Evers E. R. K. (2014). Sailing from the seas of chaos into the corridor of stability: practical recommendations to increase the informational value of studies. Pers. Psychol. 9, 278–292. doi: 10.1177/1745691614528520, PMID: 26173264

[ref78] Laukli E. Burkard R. (2015). Calibration/standardization of short-duration stimuli. Semin. Hear. 36, 03–10. doi: 10.1055/s-0034-1396923, PMID: 27516707 PMC4906301

[ref79] Lee S. K. Cha C. I. Jung T. S. Park D. C. Yeo S. G. (2008). Age-related differences in parameters of vestibular evoked myogenic potentials. Acta Otolaryngol. 128, 66–72. doi: 10.1080/00016480701387108 17851962

[ref80] Li C. Layman A. J. Carey J. P. Agrawal Y. (2015). Epidemiology of vestibular evoked myogenic potentials: data from the Baltimore longitudinal study of aging. Clin. Neurophysiol. 126, 2207–2215. doi: 10.1016/j.clinph.2015.01.008, PMID: 25703943 PMC4514573

[ref81] Lightfoot G. Sininger Y. Burkard R. Lodwig A. (2007). Stimulus repetition rate and the reference levels for clicks and short tone bursts: a warning to audiologists, researchers, calibration laboratories, and equipment manufacturers. Am. J. Audiol. 16, 94–95. doi: 10.1044/1059-0889(2007/012), PMID: 18056876

[ref82] Lipovsek M. Wingate R. J. (2018). Conserved and divergent development of brainstem vestibular and auditory nuclei. eLife 7:e40232. doi: 10.7554/eLife.40232, PMID: 30566077 PMC6317910

[ref83] Luft A. R. Skalej M. Schulz J. B. Welte D. Kolb R. Bürk K.. (1999). Patterns of age-related shrinkage in cerebellum and brainstem observed in vivo using three-dimensional MRI volumetry. Cereb. Cortex 9, 712–721. doi: 10.1093/cercor/9.7.712, PMID: 10554994

[ref84] Lütkenhöner B. (2019). Vestibular evoked Myographic correlation. J. Assoc. Res. Otolaryngol. 20, 99–114. doi: 10.1007/s10162-018-00698-9, PMID: 30421148 PMC6364267

[ref85] Macambira Y. K. D. S. Carnaúba A. T. L. Fernandes L. C. B. C. Bueno N. B. Menezes P. D. L. (2017). Aging and wave-component latency delays in oVEMP and cVEMP: a systematic review with meta-analysis. Braz. J. Otorhinolaryngol. 83, 475–487. doi: 10.1016/j.bjorl.2016.12.006, PMID: 28237301 PMC9442875

[ref86] Machino M. Ando K. Kobayashi K. Nakashima H. Tanaka S. Kanbara S.. (2021). Bioelectrical impedance analysis and manual measurements of neck circumference are interchangeable, and declining neck circumference is related to Presarcopenia. Biomed. Res. Int. 2021:6622398. doi: 10.1155/2021/6622398, PMID: 33860044 PMC8024069

[ref87] Mallik A. Weir A. I. (2005). Nerve conduction studies: essentials and pitfalls in practice. J. Neurol. Neurosurg. Psychiatry 76:ii23. doi: 10.1136/jnnp.2005.069138, PMID: 15961865 PMC1765692

[ref88] Marcus S. Whitlow C. T. Koonce J. Zapadka M. E. Chen M. Y. Williams D. W.. (2013). Computed tomography supports histopathologic evidence of vestibulocochlear sexual dimorphism. Int. J. Pediatr. Otorhinolaryngol. 77, 1118–1122. doi: 10.1016/j.ijporl.2013.04.013, PMID: 23688380

[ref89] Margolis R. H. Heller J. W. (1987). Screening tympanometry: criteria for medical referral: original papers. Audiology 26, 197–208. doi: 10.3109/00206098709081549, PMID: 3632475

[ref90] Marin-Husstege M. Muggironi M. Raban D. Skoff R. P. Casaccia-Bonnefil P. (2004). Oligodendrocyte progenitor proliferation and maturation is differentially regulated by male and female sex steroid hormones. Dev. Neurosci. 26, 245–254. doi: 10.1159/000082141, PMID: 15711064

[ref91] Mattingly J. K. Portnuff C. D. F. Hondorp B. M. Cass S. P. (2015). Sudden bilateral hearing loss after cervical and ocular vestibular evoked myogenic potentials. Eur. Acad. Otol. Neurotol. 36, 961–964. doi: 10.1097/MAO.0000000000000764 25853612

[ref92] Matuschek H. Kliegl R. Vasishth S. Baayen H. Bates D. (2017). Balancing type I error and power in linear mixed models. J. Mem. Lang. 94, 305–315. doi: 10.1016/j.jml.2017.01.001

[ref93] McNerney K. M. Burkard R. F. (2011). The vestibular evoked myogenic potential (VEMP): air- versus bone-conducted stimuli. Ear Hear. 32, e6–e15. doi: 10.1097/AUD.0b013e3182280299, PMID: 22033196

[ref94] Merchant S. N. Velázquez-Villaseñor L. Tsuji K. Glynn R. J. Wall C. Rauch S. D. (2000). Temporal bone studies of the human peripheral vestibular system. Normative vestibular hair cell data. Ann. Otol. Rhinol. Laryngol. Suppl. 181, 3–13. doi: 10.1177/00034894001090s502, PMID: 10821229

[ref95] Metwally M. I. Basha M. A. A. AbdelHamid G. A. Nada M. G. Ali R. R. Frere R. A. F.. (2021). Neuroanatomical MRI study: reference values for the measurements of brainstem, cerebellar vermis, and peduncles. Br. J. Radiol. 94:20201353. doi: 10.1259/bjr.20201353, PMID: 33571018 PMC8010561

[ref96] Moriyama H. Hayashi S. Inoue Y. Itoh M. Otsuka N. (2016). Sex differences in morphometric aspects of the peripheral nerves and related diseases. NeuroRehabilitation 39, 413–422. doi: 10.3233/NRE-161372, PMID: 27589511 PMC5008230

[ref97] Moriyama H. Itoh M. Shimada K. Otsuka N. (2007). Morphometric analysis of fibers of the human vestibular nerve: sex differences. Eur. Arch. Otorrinolaringol. 264, 471–475. doi: 10.1007/s00405-006-0197-5, PMID: 17115169

[ref98] Nakagawa S. Johnson P. C. D. Schielzeth H. (2017). The coefficient of determination R^2^ and intra-class correlation coefficient from generalized linear mixed-effects models revisited and expanded. J. R. Soc. Interface 14:20170213. doi: 10.1098/rsif.2017.0213, PMID: 28904005 PMC5636267

[ref100] Ochi K. Ohashi T. Nishino H. (2001). Variance of vestibular-evoked myogenic potentials. Laryngoscope 111, 522–527. doi: 10.1097/00005537-200103000-00025, PMID: 11224786

[ref101] Oh S.-Y. Kim H.-J. Kim J. S. (2016). Vestibular-evoked myogenic potentials in central vestibular disorders. J. Neurol. 263, 210–220. doi: 10.1007/s00415-015-7860-y, PMID: 26239221

[ref102] Oya R. Imai T. Takenaka Y. Sato T. Oshima K. Ohta Y.. (2019). Clinical significance of cervical and ocular vestibular evoked myogenic potentials in benign paroxysmal positional vertigo: a meta-analysis. Head Neck Surg. 276, 3257–3265. doi: 10.1007/s00405-019-05674-4 31605189

[ref103] Papathanasiou E. S. Murofushi T. Akin F. W. Colebatch J. G. (2014). International guidelines for the clinical application of cervical vestibular evoked myogenic potentials: an expert consensus report. Clin. Neurophysiol. 125, 658–666. doi: 10.1016/j.clinph.2013.11.042, PMID: 24513390

[ref104] Park J. H. Viirre E. (2010). Vestibular migraine may be an important cause of dizziness/vertigo in perimenopausal period. Med. Hypotheses 75, 409–414. doi: 10.1016/j.mehy.2009.04.054, PMID: 20692105

[ref105] Patterson J. N. Rodriguez A. I. Gordon K. R. Honaker J. A. Janky K. L. (2021). Age effects of bone conduction vibration vestibular-evoked myogenic potentials (VEMPs) using B81 and impulse hammer stimuli. Ear Hear. 42, 1328–1337. doi: 10.1097/AUD.0000000000001024, PMID: 33735908 PMC8387331

[ref106] Pettorossi V. E. Frondaroli A. Grassi S. (2011). Cyclic estrogenic fluctuation influences synaptic transmission of the medial vestibular nuclei in female rats. Acta Otolaryngol. 131, 434–439. doi: 10.3109/00016489.2010.536992, PMID: 21189054

[ref107] Piker E. G. Jacobson G. P. Burkard R. F. McCaslin D. L. Hood L. J. (2013). Effects of age on the tuning of the cVEMP and oVEMP. Ear Hear. 34, e65–e73. doi: 10.1097/AUD.0b013e31828fc9f2, PMID: 23673615 PMC3748259

[ref108] Portnuff C. D. F. Kleindienst S. Bogle J. M. (2017). Safe use of acoustic vestibular-evoked myogenic potential stimuli: protocol and patient-specific considerations. J. Am. Acad. Audiol. 28, 708–717. doi: 10.3766/jaaa.16071, PMID: 28906242

[ref109] R Core Team (2020). R: A language and environment for statistical Computing. Vienna: R Foundation for Statistical Computing.

[ref110] Raciti F. M. Morales Y. Snapp H. A. Rajguru S. M. (2023). A reliable and reproducible protocol for sound-evoked vestibular myogenic potentials in *rattus norvegicus*. Front. Integr. Neurosci. 17:1236642. doi: 10.3389/fnint.2023.1236642, PMID: 37731913 PMC10508189

[ref111] Raz N. Dupuis J. H. Briggs S. D. McGavran C. Acker J. D. (1998). Differential effects of age and sex on the cerebellar hemispheres and the vermis: a prospective MR study. AJNR, 19, 65–71. Available online at: https://pubmed.ncbi.nlm.nih.gov/9432159 9432159 PMC8337326

[ref112] Raz N. Gunning-Dixon F. Head D. Williamson A. Acker J. D. (2001). Age and sex differences in the cerebellum and the ventral pons: a prospective MR study of healthy adults. AJNR 22, 1161–1167. https://pubmed.ncbi.nlm.nih.gov/11415913 11415913 PMC7974784

[ref113] Raz N. Torres I. J. Spencer W. D. White K. Acker J. D. (1992). Age-related regional differences in cerebellar vermis observed in vivo. Arch. Neurol. 49, 412–416. doi: 10.1001/archneur.1992.00530280106030, PMID: 1558523

[ref114] Rhyu I. J. Cho T. H. Lee N. J. Uhm C. S. Kim H. Suh Y. S. (1999). Magnetic resonance image-based cerebellar volumetry in healthy Korean adults. Neurosci. Lett. 270, 149–152. doi: 10.1016/s0304-3940(99)00487-5, PMID: 10462116

[ref115] Rosengren S. M. Colebatch J. G. (2018). The contributions of vestibular evoked myogenic potentials and acoustic vestibular stimulation to our understanding of the vestibular system. Front. Neurol. 9:481. doi: 10.3389/fneur.2018.00481, PMID: 30013504 PMC6037197

[ref116] Rosengren S. M. Colebatch J. G. Borire A. Straumann D. Weber K. P. (2016). cVEMP morphology changes with recording electrode position, but single motor unit activity remains constant. J. Appl. Physiol. 120, 833–842. doi: 10.1152/japplphysiol.00917.2015, PMID: 26796756

[ref117] Rosengren S. M. Colebatch J. G. Young A. S. Govender S. Welgampola M. S. (2019). Vestibular evoked myogenic potentials in practice: methods, pitfalls and clinical applications. Clin. Neurophysiol. Pract. 4, 47–68. doi: 10.1016/j.cnp.2019.01.005, PMID: 30949613 PMC6430081

[ref118] Rosengren S. M. Govender S. Colebatch J. G. (2011). Ocular and cervical vestibular evoked myogenic potentials produced by air- and bone-conducted stimuli: comparative properties and effects of age. Clin. Neurophysiol. 122, 2282–2289. doi: 10.1016/j.clinph.2011.04.001, PMID: 21550301

[ref119] Rosengren S. M. Welgampola M. S. Colebatch J. G. (2010). Vestibular evoked myogenic potentials: past, present and future. Clin. Neurophysiol. 121, 636–651. doi: 10.1016/j.clinph.2009.10.016, PMID: 20080441

[ref120] Rutkove S. B. (2007). “Introduction to volume conduction” in The clinical neurophysiology primer. eds. Blum A. S. Rutkove S. B. (Cham: Springer), 43–53.

[ref121] Scarduzio M. Panichi R. Pettorossi V. E. Grassi S. (2013). Synaptic long-term potentiation and depression in the rat medial vestibular nuclei depend on neural activation of estrogenic and androgenic signals. PLoS One 8:e80792. doi: 10.1371/journal.pone.0080792, PMID: 24265837 PMC3827183

[ref122] Schumacher M. Hussain R. Gago N. Oudinet J. P. Mattern C. Ghoumari A. M. (2012). Progesterone synthesis in the nervous system: implications for myelination and myelin repair. Front. Neurosci. 6:10. doi: 10.3389/fnins.2012.00010, PMID: 22347156 PMC3274763

[ref123] Seemungal B. M. Gresty M. A. Bronstein A. M. (2001). The endocrine system, vertigo and balance. Curr. Opin. Neurol. 14, 27–34. doi: 10.1097/00019052-200102000-00005, PMID: 11176214

[ref124] Shahnaz N. David E. A. (2021). Normal values for cervical and ocular vestibular-evoked myogenic potentials using EMG scaling: effect of body position and electrode montage. Acta Otolaryngol. 141, 440–448. doi: 10.1080/00016489.2021.1887517, PMID: 33641604

[ref125] Shamim M. A. Gandhi A. P. Dwivedi P. Padhi B. K. (2023). How to perform meta-analysis in R: a simple yet comprehensive guide. Evidence 2023:1. doi: 10.61505/evidence.2023.1.1.6

[ref126] Silva T. R. Resende L. M. Santos M. A. (2016). Ocular and cervical vestibular evoked myogenic potential simultaneous in normal individuals. Codas 28, 34–40. doi: 10.1590/2317-1782/20162015040, PMID: 27074187

[ref127] Singh Ospina N. Rodriguez-Gutierrez R. Brito J. P. Young W. F. Montori V. M. (2015). Is the endocrine research pipeline broken? A systematic evaluation of the Endocrine Society clinical practice guidelines and trial registration. BMC Med. 13:187. doi: 10.1186/s12916-015-0435-z, PMID: 26265226 PMC4533940

[ref128] Smith P. F. Agrawal Y. Darlington C. L. (2019). Sexual dimorphism in vestibular function and dysfunction. J. Neurophysiol. 121, 2379–2391. doi: 10.1152/jn.00074.2019, PMID: 31042453

[ref129] Sohmer H. (2017). Soft tissue conduction: review, mechanisms, and implications. Trends Hear. 21:2331216517734087. doi: 10.1177/2331216517734087, PMID: 28969522 PMC6879487

[ref131] Straka H. Baker R. (2013). Vestibular blueprint in early vertebrates. Front. Neural Circuits 7:182. doi: 10.3389/fncir.2013.00182, PMID: 24312016 PMC3833255

[ref132] Tang B. Yu X. Jiang W. Zhang C. Zhan T. He Y. (2021). Clinical significance of serum sex hormones in postmenopausal women with vestibular migraine: potential role of estradiol. J. Int. Med. Res. 49:3000605211016379. doi: 10.1177/03000605211016379, PMID: 34024170 PMC8142534

[ref133] Thomas M. L. A. Fitzpatrick D. McCreery R. Janky K. L. (2017). Big stimulus, little ears: safety in administering vestibular-evoked myogenic potentials in children. J. Am. Acad. Audiol. 28, 395–403. doi: 10.3766/jaaa.15097, PMID: 28534730 PMC5443117

[ref134] Tiemeier H. Lenroot R. K. Greenstein D. K. Tran L. Pierson R. Giedd J. N. (2010). Cerebellum development during childhood and adolescence: a longitudinal morphometric MRI study. NeuroImage 49, 63–70. doi: 10.1016/j.neuroimage.2009.08.016, PMID: 19683586 PMC2775156

[ref135] Tourtillott B. M. Ferraro J. A. Bani-Ahmed A. Almquist E. Deshpande N. (2010). Age-related changes in vestibular evoked myogenic potentials using a modified blood pressure manometer feedback method. Am. J. Audiol. 19, 100–108. doi: 10.1044/1059-0889(2010/10-0021), PMID: 20966352

[ref136] Vasavada A. N. Danaraj J. Siegmund G. P. (2008). Head and neck anthropometry, vertebral geometry and neck strength in height-matched men and women. J. Biomech. 41, 114–121. doi: 10.1016/j.jbiomech.2007.07.007, PMID: 17706225

[ref137] Velázquez-Villaseñor L. Merchant S. N. Tsuji K. Glynn R. J. Wall C. Rauch S. D. (2000). Temporal bone studies of the human peripheral vestibular system. Normative Scarpa’s ganglion cell data. Ann. Otol. Rhinol. Laryngol. Suppl. 181, 14–19. doi: 10.1177/00034894001090s503, PMID: 10821230

[ref138] Viechtbauer W. (2010). Conducting meta-analyses in R with the metafor package. J. Stat. Softw. 36, 1–48. doi: 10.18637/jss.v036.i03

[ref140] Wei W. Jeffcoat B. Mustain W. Zhu H. Eby T. Zhou W. (2013). Frequency tuning of the cervical vestibular-evoked myogenic potential (cVEMP) recorded from multiple sites along the sternocleidomastoid muscle in normal human subjects. J. Assoc. Res. Otolaryngol. 14, 37–47. doi: 10.1007/s10162-012-0360-1, PMID: 23183876 PMC3540270

[ref141] Welgampola M. S. Colebatch J. G. (2001). Vestibulocollic reflexes: normal values and the effect of age. Clin. Neurophysiol. 112, 1971–1979. doi: 10.1016/S1388-2457(01)00645-9, PMID: 11682335

[ref142] Wiener-Vacher S. R. Campi M. Boizeau P. Thai-Van H. (2023). Cervical vestibular evoked myogenic potentials in healthy children: normative values for bone and air conduction. Front. Neurol. 14:1157975. doi: 10.3389/fneur.2023.1157975, PMID: 37143993 PMC10152971

[ref143] Williams M. Howell P. (1990). An electrical network model of inertially induced bone-conducted sound. Scand. Audiol. 19, 161–170. doi: 10.3109/01050399009070767, PMID: 2237255

[ref144] Winter B. (2019). Statistics for linguists: an introduction using R. Available online at: http://books.google.co.uk/books?id=uVNGxQEACAAJ&hl=&source=gbs_api

[ref145] Wit H. P. Kingma C. M. (2006). A simple model for the generation of the vestibular evoked myogenic potential (VEMP). Clin. Neurophysiol. 117, 1354–1358. doi: 10.1016/j.clinph.2006.03.014, PMID: 16678484

[ref146] Yeo S. W. Chang K. H. Park S. N. Suh B. D. (2003). Effects of testosterone in the treatment of immune-mediated sensorineural hearing loss. Eur. Arch. Otorrinolaringol. 260, 316–319. doi: 10.1007/s00405-002-0570-y, PMID: 12883955

